# Allele-specific mitochondrial stress induced by Multiple Mitochondrial Dysfunctions Syndrome 1 pathogenic mutations modeled in *Caenorhabditis elegans*

**DOI:** 10.1371/journal.pgen.1009771

**Published:** 2021-08-27

**Authors:** Peter A. Kropp, Jing Wu, Michael Reidy, Sanjay Shrestha, Kyle Rhodehouse, Philippa Rogers, Michael N. Sack, Andy Golden

**Affiliations:** 1 Laboratory of Biochemistry and Genetics, National Institute of Diabetes and Digestive and Kidney Diseases, National Institutes of Health, Bethesda, Maryland, United States of America; 2 Mitochondrial Biology and Metabolism, Cardiovascular Branch, National Heart, Lung, and Blood Institute, National Institutes of Health, Bethesda, Maryland, United States of America; Case Western Reserve University School of Medicine, UNITED STATES

## Abstract

Multiple Mitochondrial Dysfunctions Syndrome 1 (MMDS1) is a rare, autosomal recessive disorder caused by mutations in the *NFU1* gene. NFU1 is responsible for delivery of iron-sulfur clusters (ISCs) to recipient proteins which require these metallic cofactors for their function. Pathogenic variants of *NFU1* lead to dysfunction of its target proteins within mitochondria. To date, 20 *NFU1* variants have been reported and the unique contributions of each variant to MMDS1 pathogenesis is unknown. Given that over half of MMDS1 individuals are compound heterozygous for different *NFU1* variants, it is valuable to investigate individual variants in an isogenic background. In order to understand the shared and unique phenotypes of *NFU1* variants, we used CRISPR/Cas9 gene editing to recreate exact patient variants of *NFU1* in the orthologous gene, *nfu-1* (formerly *lpd-8)*, in *C*. *elegans*. Five mutant *C*. *elegans* alleles focused on the presumptive iron-sulfur cluster interaction domain were generated and analyzed for mitochondrial phenotypes including respiratory dysfunction and oxidative stress. Phenotypes were variable between the mutant *nfu-1* alleles and generally presented as an allelic series indicating that not all variants have lost complete function. Furthermore, reactive iron within mitochondria was evident in some, but not all, *nfu-1* mutants indicating that iron dyshomeostasis may contribute to disease pathogenesis in some MMDS1 individuals.

## Author summary

Functional mitochondria are essential to life in eukaryotes, but they can be perterbured by inherent dysfunction of important proteins or stressors. Mitochondrial dysfunction is the root cause of dozens of diseases many of which involve complex phenotypes. One such disease is Multiple Mitochondrial Dysfunctions Syndrome 1, a pediatric-fatal disease that is poorly understood in part due to the lack of clarity about how mutations in the causative gene, *NFU1*, affect protein function and phenotype development and severity. Here we employ the power of CRISPR/Cas9 gene editing in the small nematode *Caenorhabditis elegans* to recreate five patient-specific mutations known to cause Multiple Mitochondrial Dysfunctions Syndrome 1. We are able to analyze each of these mutations individually, evaluate how mitochondrial dysfunction differs between them, and whether or not the phenotypes can be improved. We find that there are meaningful differences between each mutation which not only effects the types of stress that develop, but also the ability to rescue deleterious phenotypes. This work thus provides insight into disease pathogenesis and establishes a foundation for potential future therapeutic intervention.

## Introduction

Mitochondrial diseases pose a unique challenge to both clinicians and researchers due to the diverse and sometimes inconsistent symptoms. However, much progress has been made in recent years to better understand how specific types of mitochondrial dysfunction present in both patients and model organisms. Clarification of mitochondrial pathways provides the opportunity to better understand disease pathogenesis and potentially develop therapeutics for mitochondrial disease. Because of the interconnection between mitochondrial pathways, investigation into one factor or pathway has the potential to provide insight into a broader array of biological processes and functions.

One such pathway that has far-reaching effects is iron-sulfur cluster biogenesis. Iron-sulfur clusters (ISCs) are ancient metallic cofactors found in approximately 70 human proteins [1]. While ISC-containing proteins (ISPs) are found throughout the cell, they are concentrated within the mitochondria [1]. This pathway contains two distinct phases: an initial biogenesis phase that generates the ISCs and a maturation/delivery phase that delivers the ISCs to recipient proteins (reviewed in [2,3]). Although the traditional view restricts the biogenesis phase to the mitochondrial matrix, recent data has indicated that *de novo* ISC biogenesis can occur in either the mitochondrial matrix or cytosol [4]. Likewise, the maturation/delivery phase can occur via highly related but independent processes either in the mitochondrial matrix or the cytosol. Aspects of both delivery processes remain unclear. Within the mitochondrial process, specific delivery proteins bind to either 2Fe-2S or 4Fe-4S ISCs and deliver them to the appropriate recipient. The best understood of these ISC delivery proteins is NFU1.

NFU1 binds 4Fe-4S ISCs and delivers them to targets such as lipoic acid synthase (LIAS), aconitase, and succinate dehydrogenase [5–11]. NFU1 also plays a role in delivery of ISCs to components of complex I of the electron transport chain (ETC), yet the details of this delivery remain to be clarified [12,13]. The function of NFU1 to deliver ISCs to its targets is of vital importance as variations altering the function of NFU1 are causative in the disease Multiple Mitochondrial Dysfunctions Syndrome 1 (MMDS1) [14–28]. MMDS1 is autosomal recessive requiring two variant alleles for the disease to be pathogenic. Approximately 50% of MMDS1-affected individuals do not carry homozygous variants of *NFU1*. Rather, they are compound heterozygous and carry two different variant *NFU1* alleles making the pathogenicity of each individual variant difficult to discern. There have been 20 variant *NFU1* alleles reported at the time of writing. Consistent with many mitochondrial diseases, MMDS1 has diverse and inconsistent symptoms. Tragically, the most consistent aspect of MMDS1 is pediatric lethality with almost all patients passing away before they are 2-years old [14–19,21–24,26]. These individuals typically develop normally for a period of time before rapid neurological regression, reduced or lost motor function, and development of pulmonary hypertension. The observed neurological regression is likely due to brain lesions including leukoencephalopathy, a common finding in the majority of patients [16–18,20–23,26].

Biochemically, MMDS1 individuals frequently present with hyperglycinemia, lactic acidosis, reduced ETC function, and reduced pyruvate dehydrogenase function [14–23,25,26]. Many of these symptoms can be attributed, directly or indirectly, to dysfunction of the five oxoacid dehydrogenases that are dependent upon lipoic acid for their function. Therefore, arguably the most important effect of reduced NFU1 activity is the loss of ISC delivery to LIAS which requires ISC delivery by NFU1 to generate lipoic acid [6,7]. Amongst these lipoylated oxoacid dehydrogenases are the E2 subunits of the pyruvate dehydrogenase (PDH) complex, the α-ketoglutarate dehydrogenase (KGDH) complex, and the H protein of the glycine cleavage system [29,30]. It is thus unsurprising that MMDS1 individuals present with reduced PDH function and hyperglycinemia. Although the biochemical effects of dysfunction of lipoylated proteins are known [29], it remains unclear how the pathogenic variations in NFU1 differentially affect LIAS and broader mitochondrial and cellular function. Utilization of multicellular model systems is a promising means to better understand the pathogenicity of MMDS1-associated mutations.

The nematode *Caenorhabditis elegans* (*C*. *elegans*) is one of the smallest multicellular model organisms but one for which there is a fully sequenced genome, invariant and fully defined somatic cell lineage, and cell biology that is nearly identical to humans. *C*. *elegans* has proven a powerful tool for understanding human disease biology. Manipulations of gene function, either with RNA interference (RNAi) or gene editing, is facile in *C*. *elegans* allowing for in depth studies of gene function and genetic interactions with other factors. Furthermore, *C*. *elegans* has been instrumental in understanding aging and stress pathways that are extremely well conserved across organisms and highly relevant to human disease [31–33]. One of these pathways, insulin and insulin-like signaling, is regulated by the transcription factor DAF-16. DAF-16 is capable of responding to multiple forms of stress (e.g. oxidative or nutrient stress) to activate transcriptional programs necessary for stress resistance and longevity [34–37]. Of particular interest to this study, DAF-16 also positively regulates the *C*. *elegans* response to reactive iron [38].

In this study, we used CRISPR/Cas9 gene editing approaches to recreate select MMDS1 patient-specific variants in NFU-1 (encoded by the gene *nfu-1*, formerly known as *lpd-8*), the orthologous protein to human NFU1. We generated five alleles focused on mutations of residues that disrupt the presumptive ISC-interaction domain. We have found that these variants consistently present with mitochondrial homeostasis defects but also with notable differences in severity and presentation of specific phenotypes between the alleles. Mutations closest to the canonical CXXC ISC-binding motif have the most severe effects and recapitulate many of the same dysfunctions present in MMDS1 individuals. Furthermore, we identify activation of the DAF-16 stress response pathway as the primary means of compensation to mitochondrial stress with elevated reactive iron and respiratory defects as the primary effectors.

## Results

### *nfu-1/lpd-8* is orthologous to *NFU1*

**Fig 1:**
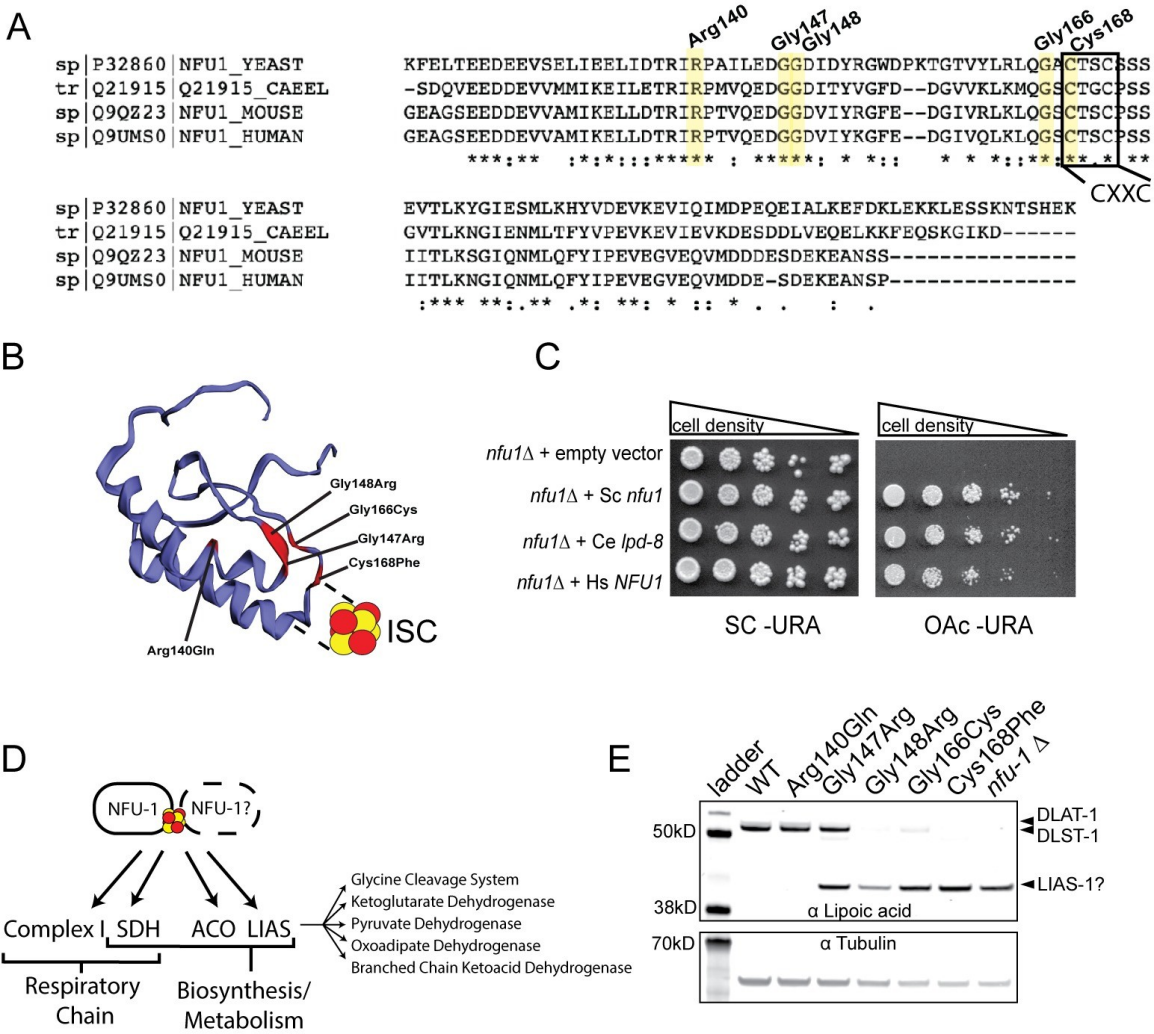
*C*. *elegans nfu-1/lpd-8* is orthologous to human and yeast *NFU1*. (A) Partial Clustal Omega alignment of yeast Nfu1, *C*. *elegans* NFU-1/LPD-8, mouse Nfu1, and human NFU1 highlighting five residues mutated in MMDS1 and the conserved CXXC ISC-interaction motif. (B) Predicted structure of the *C*. *elegans* NFU-1 C-terminal domain with mutated residues annotated in red and labeled with corresponding mutations. (C) Representative complementation assay with yeast *nfu1*, *C*. *elegans nfu-1/lpd-8*, and human *NFU1* on complete (SC) and acetate (OAc) medium. Cell density indicated above images. (D) Known targets of NFU1/NFU-1 and downstream pathways/complexes affected. NFU1/NFU-1 predominantly functions as a homodimer, but other binding partners are possible (indicated by dotted line and question mark) (E) Representative western blot analysis of lipoylated proteins from L4 WT and *nfu-1* mutants. Lipoylated proteins are above and tubulin (loading control) is below. DLAT-1 (E2 subunit of PDH; 53kD) and DLST-1 (E2 subunit of KGDH; 49.8kD) indicated to the right. The lower band is consistent with LIAS-1 (39.8kD) but could not be verified.

The *C*. *elegans* gene *lpd-8* produces the protein LPD-8 with 43.6% amino acid similarity to human NFU1 (UniProt). Sequence similarity is highest in the C-terminal domain, which, in both proteins, contains the canonical CXXC ISC binding motif (Fig 1A and 1B). Notably, 29 of 37 MMDS1 individuals carry at least one mutation to either this motif or adjacent residues. To confirm that LPD-8 is orthologous to NFU1, a complementation assay was performed in the budding yeast *Saccharomyces cerevisiae*, which is also known to have the conserved NFU1 orthologue Nfu1 [8,39]. On a non-fermentable carbon source, LPD-8 was capable of complementing lost Nfu1 function in *nfu1Δ* yeast equally as well as human NFU1, demonstrating that they are functionally orthologous (Fig 1C). Therefore, with the approval of WormBase, *lpd-8* will henceforth be referred to as *nfu-1*. Within the C-terminal domain of both human NFU1 and *C*. *elegans* NFU-1, the residues affected by pathogenic mutations and resulting in MMDS1 are all well conserved (Fig 1A). We chose to focus on five residues within the presumptive ISC-binding motif that are documented to be mutated in MMDS1 individuals. CRISPR/Cas9 genome editing was used to recreate these variants in *nfu-1*/NFU-1 (Table 1) in addition to a full *nfu-1* deletion (*nfu-1*Δ). Each of these variants was analyzed in homozygous animals allowing for the elucidation of unique phenotypes caused by each variation. Note, the nomenclature associated with the amino acid change will be used throughout this manuscript rather than the *C*. *elegans* allele or strain name in order to better illustrate the unique differences between the mutants.

**Table 1:** Summary of MMDS1 alleles modeled in this study.

MMDS1 mutation	*C*. *elegans* mutation
Arg182Gln	Arg140Gln
Gly189Arg	Gly147Arg
Gly190Arg	Gly148Arg
Gly208Cys	Gly166Cys
Cys210Phe	Cys168Phe

Most of the *nfu-1* mutations resulted in impaired delivery of lipoic acid to PDH and KGDH (Fig 1D and 1E) as has also been observed in MMDS1 samples [16,19], patient-derived fibroblasts [11,15,17,19,21,28,40], and NFU1 mutant rodents [41], plants [42], and yeast [8]. A single mutant, Arg140Gln appears unaffected in its ability to lipoylate PDH and KDGH. The Gly147Arg mutant appears to have impaired, although not abolished, lipoylation of PDH and KDGH whereas all other mutations and the *nfu-1*Δ demonstrate severe reductions in lipoylation (Fig 1E). Interestingly, a lower molecular weight band was detected in some *nfu-1* mutants that appears to be the lipoate or octanoyl moiety retained within LIAS (LIAS-1 in *C*. *elegans*) (Fig 1E). LIAS requires a second ISC, delivered by NFU1, for the conversion of the octanoyl moiety to a fully-formed lipoic acid [6,7], which is necessary for lipoylation of target proteins. This lower band is consistent with the molecular weight of *C*. *elegans* LIAS-1, but we were unable to confirm this band’s identity as the available LIAS antibody did not work in our assays. Retention of the octanoyl moiety within LIAS has not been reported before. As will be discussed more thoroughly in the Discussion, the lack of a protein lipoylation defect in the Arg140Gln mutant is not wholly surprising. In humans, this mutation also disrupts a splice site ultimately resulting in NFU1 protein degradation [15,21]. As the *nfu-1* gene structure is not conserved, no splice sites are near the mutated codon that results in the Arg140Gln change and thus a protein degradation phenotype seems unlikely. Despite attempts with multiple antibodies, we were unable to convincingly or reliably measure NFU-1 protein in *C*. *elegans* samples, however, mRNA levels were unchanged in the *nfu-1* mutants (S1 Fig). Therefore, the phenotypes presented herein are likely due to the amino acid changes of the individual mutants.

### *nfu-1* variants display characteristic phenotypes of mitochondrial dysfunction

*C*. *elegans* mitochondrial mutants characteristically present with phenotypes associated with oxidative and/or nutrient stress such as sterility, mitohormesis (protection from exogenous stress), and extended lifespan in spite of typically arresting at the L3 stage [43]. Homozygous *nfu-1* mutants did not arrest as L3 larvae, but rather the most severely affected mutants arrested as early L4 larvae (Gly148Arg, Gly166Cys, Cys168Phe and *nfu-1*Δ) or late L4/young adult (Gly147Arg). Not only did these mutants arrest as larvae, they also displayed delayed maturation, taking ~18–24 hours longer than wild-type (WT) to reach the L4 stage. The Arg140Gln mutant did not arrest and was not delayed in development. Because of this growth arrest in most mutant strains, all assays described herein were performed on homozygous populations of mutant animals as soon as they reached the L4 stage. This arrest was consistent with RNAi knockdown of *nfu-1* although the RNAi effect was not as severe as the CRISPR mutants (S2A–S2C Fig).

**Fig 2:**
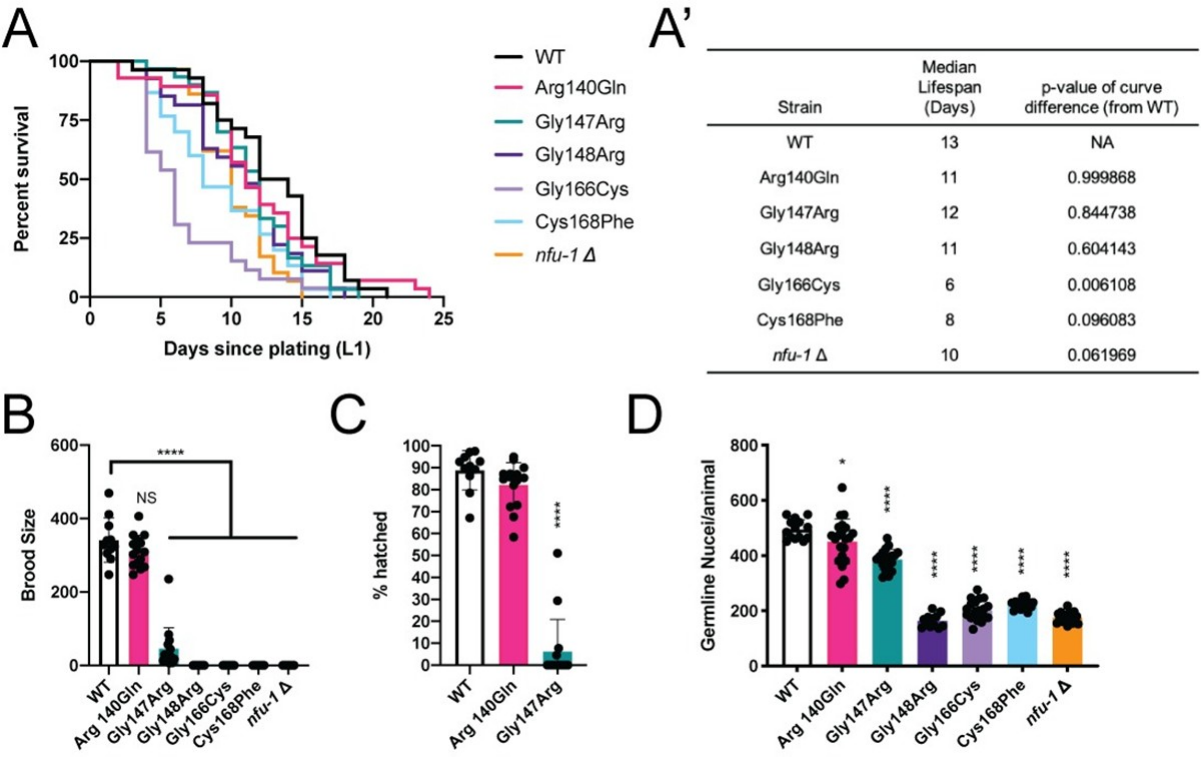
Mutations to *nfu-1* affect lifespan and fecundity. (A-A’) Kaplan-Meier survival curve and analysis of *nfu-1* mutants (n = 26–30). (B) Total brood size (n = 11–15 broods), (C) embryonic viability (n = 11–15 broods), and (D) L4 germline nuclei of *nfu-1* mutants (n = 12–22). A’: Log-rank (Mantle-Cox) analysis with Bonferroni correction for multiple comparisons. B-D: *: p≤0.05; ****: p≤0.0001 difference between WT and mutant by One-way ANOVA with Dunnett correction for multiple comparisons. NS: Not significant.

Despite larval arrest, many *C*. *elegans* mitochondrial mutant strains have extended lifespans [43,44]. While the exact mechanism for this extension has been debated, it appears that moderate mitochondrial stress, from reactive oxygen species (ROS) or otherwise, activates longevity pathways, especially those regulated by the FOXO transcription factor DAF-16 [35]. However, severe mitochondrial stress including knockdown of ETC subunits can shorten lifespan [44]. None of the *nfu-1* mutants had extended lifespans though some did have shorter lifespans than WT animals indicating more severe mitochondrial stress (Fig 2A and 2A’). The most dramatic reduction was in the Gly166Cys mutant. Although not statistically significant, the Cys168Phe and *nfu-1*Δ mutants trended toward shortened lifespans. This reduced viability is also supported by RNAi knockdown of *nfu-1* which was lethal in ~50% of animals within 72 hours of being laid (S2D Fig).

Amongst the *nfu-1* mutants, all but the Arg140Gln mutant were sterile (Fig 2B and 2C). The Gly147Arg mutant was inconsistently capable of laying fertilized embryos, but only a small percentage of homozygous animals did so and the progeny laid were rarely viable (Fig 2B and 2C). Furthermore, Gly147Arg mutants laid those few progeny at a delayed time frame compared to WT (S2E Fig). Due to this sterility, all *nfu-1* mutant strains (except Arg140Gln) were maintained as balanced heterozygotes but analyzed as homozygotes by manual picking of homozygous animals from a mixed population. The observed sterility was in part due to reduced germline size, as evidenced by fewer nuclei within the germline of L4 animals (Fig 2D). Specification of the germline (presence of both Z2 and Z3 cells in L1 larvae) was unimpaired and the reduction in germline nuclei appears to be due to increased apoptosis of germ cells (S2F Fig). Further, other than the Arg140Gln and Gly147Arg mutants, *nfu-1* mutants did not produce mature gametes. Notably, the observed sterility appears to be largely, although not exclusively, cell-autonomous as germline-specific RNAi knockdown of *nfu-1* had substantially increased sterility whereas knockdown in somatic tissues had limited or no effect (S2G Fig).

### Disrupted mitochondrial physiology in *nfu-1* mutant alleles

**Fig 3:**
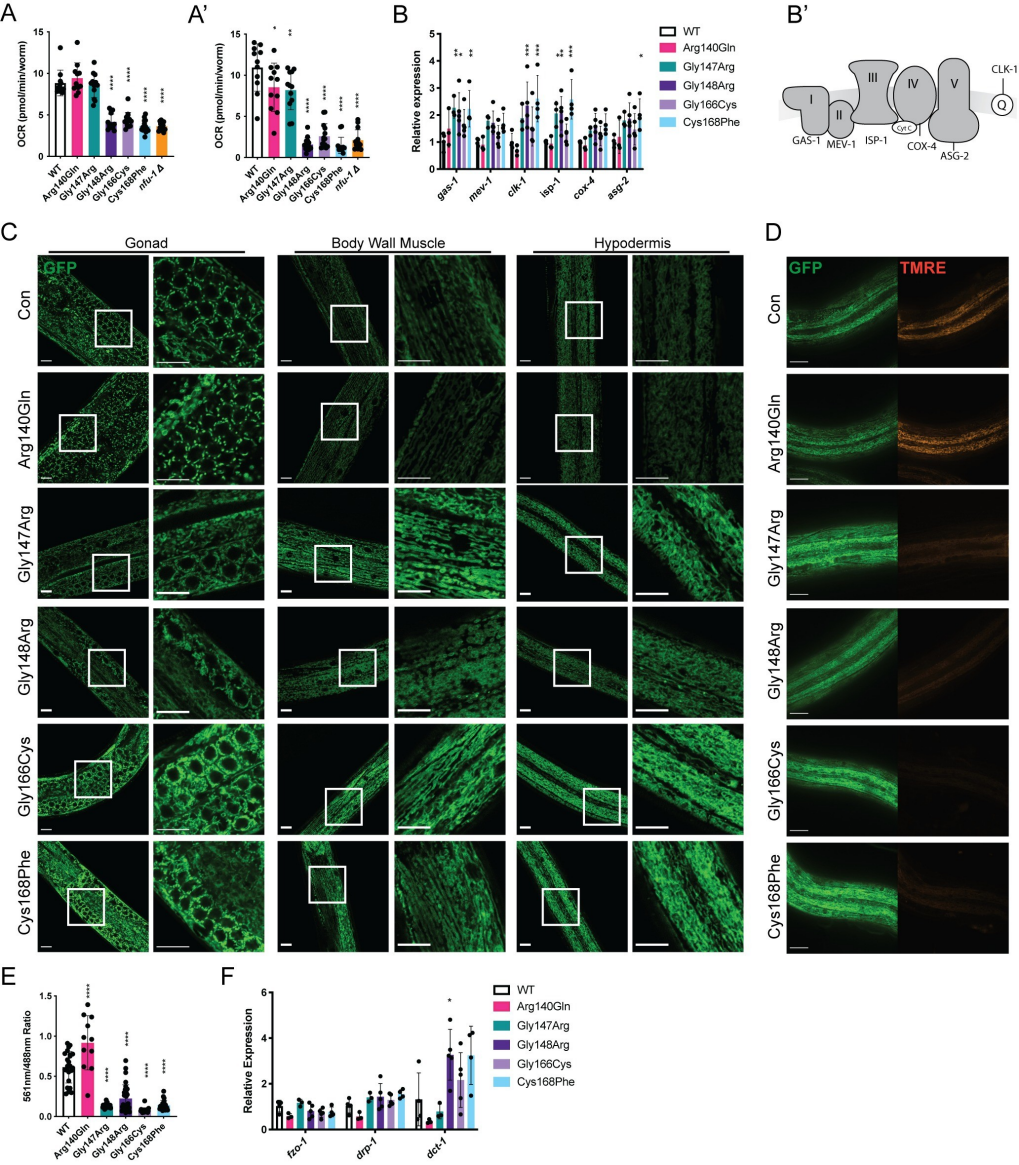
Mitochondrial physiology and function are impaired by *nfu-1* mutations. (A-A’) OCR as measured via Seahorse XFe96 (n = 11–14). (A) Basal OCR. (A’) Maximal OCR. (B) Gene expression of ETC components and accessory factors (n = 3–5). Genes assessed in order presented: *gas-1* (CI), *mev-1* (CII), *clk-1* (Coenzyme Q), *isp-1* (CIII), *cox-4* (CIV), and *asg-2* (CV). (B’) ETC schematic with the factor measured in (B) indicated for each complex or accessory factor. (C) Representative COX-4::GFP expression in mitochondria of WT and *nfu-1* mutants in the gonad, body wall muscle, and hypodermis. Images captured at 63X or 63X + 3X digital zoom. White boxes indicate regions enhanced at right. Green: COX-4::GFP. Scale bar: 10μm. (D) TMRE labeling of hypodermal mitochondria in WT and *nfu-1* mutants. Green: COX-4::GFP; Red: TMRE. Scale bar: 20μm. (E) Quantification of (D). Ratiometric measure of pixel intensity (561nm/488nm) (n = 11–32). (F) Gene expression mitochondrial fission (*fzo-1*), fusion (*drp-1*) and mitophagy (*dct-1*) positive regulators (n = 3–5). A-B; E-F: *: p≤0.05; **:p≤0.01; ***:p≤0.001; ****:p≤0.0001 difference between control and *nfu-1* mutant by One-way ANOVA with Dunnett correction for multiple comparisons. OCR: oxygen consumption rate; ETC: electron transport chain; TMRE: Tetramethylrhodamine, Ethyl Ester.

The reduced fertility, larval arrest, and altered lifespan observed in the more severe *nfu-1* mutants are consistent with mitochondrial dysfunction, so the ability of these *nfu-1* mutants to effectively perform oxidative phosphorylation (OxPhos) was assessed. OxPhos, alternatively called aerobic respiration, terminates with ATP production by ATP synthase following the electron transfer down the ETC and the consumption of reducing equivalents as well as oxygen by complexes I through IV. Oxygen consumption rates (OCRs), a measure of ETC function, can be accurately assessed using a Seahorse XFe96 bioanalyzer with intact *C*. *elegans* [45,46]. Both basal and maximal OCRs were severely, and comparably, reduced in the Gly148Arg, Gly166Cys, Cys168Phe and *nfu-1*Δ mutants (Figs 3A, 3A’, and S3A) indicating impaired ETC function. The Arg140Gln and Gly147Arg mutants both had normal basal OCR with slight but significant reductions in maximal OCR that are of equal magnitude (Figs 3A, 3A’, and S3A). Given that the Arg140Gln mutant is fertile and the Gly147Arg mutant is effectively sterile, these data suggest that a stressor other than ETC dysfunction is likely the primary cause of sterility in the Gly147Arg mutant. These data collectively indicate that mutations affecting the ISC interaction domain of NFU-1 impact OxPhos differently depending on the mutation.

As the Gly148Arg and Cys168Phe mutants are indistinguishable from the *nfu-1*Δ mutant in all measures thus far, we consider these mutations to be loss-of-function alleles. Additionally, the Cys168Phe mutation directly abolishes the first cysteine of the CXXC motif necessary to bind an ISC, which should lead to a complete loss of function. For these reasons the *nfu-1*Δ strain was not included in further analyses.

Reasons for dysfunctional OxPhos in *nfu-1* mutants can likely be attributed to impaired or absent function of the ISP targets of NFU-1. Studies in yeast and human fibroblasts have shown that NFU1 contributes to the delivery of ISCs to Complex I (CI) of the ETC [12,13] and directly delivers an ISC to succinate dehydrogenase B (SDHB), a component of CII of the ETC [8,14–21] (Fig 1D). Therefore, the Seahorse results indicating ETC dysfunction are not surprising. A common cellular response to dysfunction within the ETC is to up-regulate the expression of the individual components [44,47,48]. Quantitative reverse transcription PCR (qRT-PCR) was used to measure gene expression of essential components of each complex of the ETC as well as the electron carrier coenzyme Q (Q). The Gly148Arg and Cys168Phe mutants consistently up-regulated ETC components (Fig 3B and 3B’). Although the Gly147Arg and Gly166Cys mutants consistently trended toward increased gene expression, these changes were rarely statistically significant. Among the mutant strains, up-regulation in genes associated with CI, Q, and CIII were most consistently observed, suggesting attempted compensation for dysfunction in the early steps of the ETC. This conclusion is supported by Seahorse data that inhibition of Complex IV of the ETC resulted in equivalent decreases in OCR from basal in all genotypes (S3B Fig). Collectively, these data indicate that those *nfu-1* mutants with impaired ETC function also typically compensate through up-regulation of ETC components.

**Fig 4:**
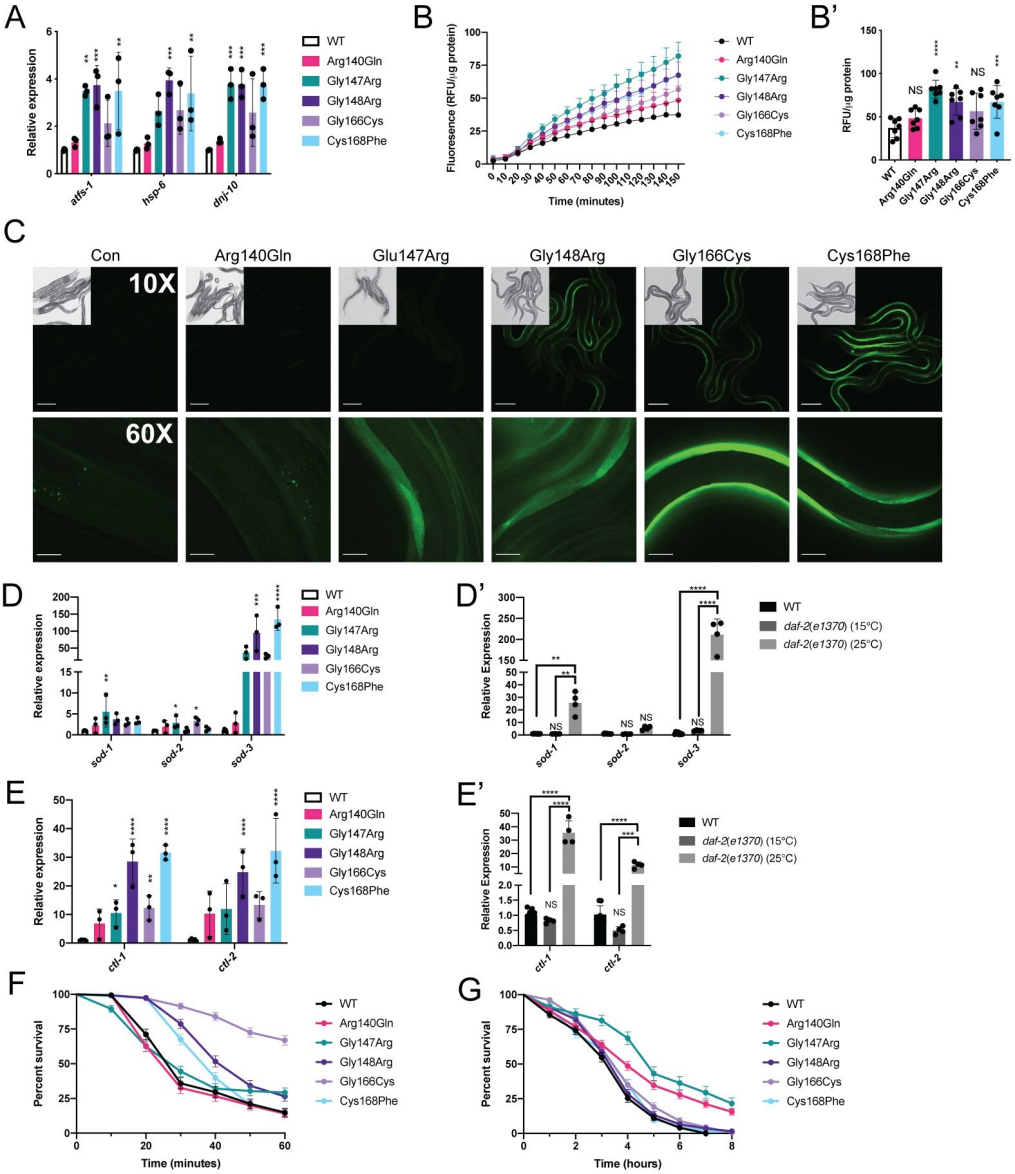
Activation of multiple stress response pathways in *nfu-1* mutants. (A) Gene expression of *atfs-1* and direct targets in the UPR^mt^ (n = 3). (B-B’) H_2_DCFDA fluorescent signal for ROS (n = 7). (B) Fluorescent signal over time. (B’) End point fluorescent signal. (C) Representative images of the *Pgst-4::gst-4::GFP* transgene in control (transgene only) and *nfu-1* mutants. Scale bar: 100μm in 10X; 20μm in 60X images. Insets at 10X show DIC image of animals in the field of view. (D-D’) Gene expression of superoxide dismutase genes (n = 3–4). (E-E’) Gene expression of catalase genes (n = 3–4). (F, G) Kaplan-Meier survival curves with exposure to exogenous oxidants. (F) H_2_O_2_ exposure (n = 134–175). (G) Paraquat exposure (n = 102–251). Data in (F-G) represented as percentages ± standard error. Statistical analysis in Table 2. *: p≤0.05; **:p≤0.01; ***:p≤0.001; ****:p≤0.0001 difference between (A-B,D,E) WT and *nfu-1* mutants by One-way ANOVA with Dunnett correction for multiple comparisons; (D’,E’) all groups by One-way ANOVA with Tukey correction for multiple comparisons. NS: Not significant. RFU: Relative fluorescence units.

As the previous assays investigated whole-animal mitochondrial function, it remained unclear how mitochondrial stress in individual tissues was affected in the *nfu-1* mutants. Analysis of mitochondrial shape and size can provide an indication of mitochondrial homeostasis. A COX-4::GFP fusion protein [49] was used to analyze mitochondrial structure in the gonad, body wall muscle, and hypodermis of the *nfu-1* mutants. This analysis revealed altered mitochondrial morphology in all *nfu-1* mutants except the Arg140Gln mutant. Namely, mitochondria appeared enlarged or swollen compared to WT in all tissues analyzed, consistent with mitochondrial stress (Fig 3C). We believe that the enlarged mitochondria are responsible for the apparent increase in fluorescence intensity observed in most tissues. However, the elevated expression of ETC components (Fig 3B) did suggest that there could be increased mitochondrial biogenesis in the more severe *nfu-1* mutants. In *C*. *elegans*, mitochondrial biogenesis is largely regulated by the NRF2 ortholog SKN-1 [50]. As will be shown in Fig 4, SKN-1 is activated in the *nfu-1* mutants. However, assessment of mitochondrial DNA content indicated no change in the *nfu-1* mutants compared to WT with the exception of a slight but significant increase in the Gly147Arg mutant (S3C Fig). Therefore, increased mitochondrial biogenesis is unlikely in the more severe *nfu-1* mutants.

Evidence of mitochondrial stress was further supported by reduced labeling of mitochondria with tetramethylrhodamine (TMRE), a dye that accumulates in mitochondria with a hyperpolarized mitochondrial membrane potential (Δψ^m^) (Figs 3D, 3E, and S3E). Under normal homeostatic conditions, Δψ^m^ is maintained by the ETC’s establishment of a proton gradient. As expected, the mutants with decreased OCR also had significantly decreased TMRE accumulation (Figs 3D, 3E, and S3E). Both the Arg140Gln and Gly147Arg mutants presented with surprising TMRE findings. The Arg140Gln mutant demonstrated increased TMRE accumulation whereas the Gly147Arg mutant had significantly reduced TMRE labeling in spite of the fact that basal OCR was unchanged in either mutant. This result suggests that a stressor other than ETC dysfunction was reducing Δψ^m^ in the Gly147Arg mutant. The altered TMRE labeling relative to COX-4::GFP was not an artifact of increased COX-4::GFP signal intensity (S3D Fig) or increased *cox-4* gene expression (Fig 3B).

In addition to mitochondrial biogenesis, the possibility of altered mitochondrial fission or fusion was explored. Gene expression of the mitochondrial fission (*fzo-1*) or fusion (*drp-1*) regulators was unchanged (Fig 3F) suggesting that these processes are not active in the *nfu-1* mutants. However, the mitophagy regulator *dct-1* was significantly elevated in the Gly148Arg mutant (Fig 3F) suggesting increased mitophagy in this mutant. Although statistical significance was not reached, both the Gly166Cys and Cys168Phe mutants also trended toward elevated *dct-1* expression with the magnitude of the increase in the Cys168Phe mutant being equal to the Gly148Arg mutant. However, the Gly148Arg mutant strain is the only one that presented with a mitochondrial morphology phenotype (fragmentation) potentially indicative of mitophagy, especially in the body wall muscle (Fig 3C). Collectively, these data indicate that all *nfu-1* mutants, to varying degrees, have altered mitochondrial physiology. In the case of the Gly148Arg, Gly166Cys, and Cys168Phe mutants, the results are largely consistent with ETC dysfunction; however, this was not true for the *nfu-1* Gly147Arg mutant. A different stressor, possibly oxidative stress, appears to disrupt both mitochondrial morphology and Δψ^m^ in this mutant.

### Mitochondrial stress responses are activated in *nfu-1* mutants

*C*. *elegans* have multiple pathways by which they can respond to stressors including three canonical pathways to respond to mitochondrial stress. The first is the mitochondrial unfolded protein response (UPR^mt^) which is mediated by the transcription factor ATFS-1 (orthologue of mammalian ATF5). ATFS-1 is normally imported into the mitochondria and degraded by the protease LON [51,52]. If Δψ^m^ is not maintained, ATFS-1 cannot be imported into mitochondria and it instead localizes to the nucleus and activates genes responsible for remodeling mitochondrial proteostasis and metabolism [51,52]. The second and third pathways, mediated by the transcription factors SKN-1 and DAF-16, respectively, can both be directly activated by oxidative stress. SKN-1 (orthologue of mammalian NRF2), is tonically expressed but normally proteasomally degraded [53]. When released from its degradation cycle, SKN-1 is transported to the nucleus where it activates a transcriptional program associated with ROS detoxification and metabolic remodeling, especially fatty acid elongation [54–56]. DAF-16 (orthologue of mammalian FOXO) is also tonically expressed but normally sequestered in the cytoplasm by heatshock proteins [31]. When released from sequestration it translocates to the nucleus where it activates a transcriptional program also associated with ROS detoxification and metabolic remodeling, especially fatty acid oxidation [34]. While each of these pathways can be individually activated, they have significant overlap in activating stimuli (such as ROS) and downstream targets (such as ROS detoxification genes). Furthermore, the transcription factors may be dependent on each other in specific contexts such as during development [57].

It was first determined whether the UPR^mt^ was activated in many of the *nfu-1* mutants as would be expected in the strains with impaired Δψ^m^ (Figs 3D, 3E, and S3E). The possibility of UPR^mt^ activation was first assessed with a GFP reporter strain (*hsp-6p::GFP* [58]) exposed to *nfu-1* RNAi. Knockdown of *nfu-1* activated this reporter to nearly the same extent as the control RNAi, *spg-7*, indicating that reduced NFU-1 expression or function is sufficient to activate the UPR^mt^ (S4A Fig). Activation of the UPR^mt^ was next assessed in the individual *nfu-1* mutants via analysis of known ATFS-1 target genes. ATFS-1 positively regulates itself as well as targets related to mitochondrial proteostasis including *hsp-6* and *dnj-10* [52,59]. As expected, *atfs-1* mRNA was significantly increased in the Gly147Arg, Gly148Arg, and Cys168Phe animals, as were its targets *hsp-6* and *dnj-10* (Fig 4A). Surprisingly, mRNA expression of each of these genes trended towards an increase in the Gly166Cys mutant but did not reach statistical significance (Fig 4A). These data indicated that, in spite of decreased Δψ^m^ in the Gly147Arg, Gly148Arg, Gly166Cys, and Cys168Phe mutants, activation of the UPR^mt^ was unequal suggesting more complicated sources of mitochondrial stress.

Oxidative stress is sufficient for the activation of both SKN-1 and DAF-16, so it was next determined whether the *nfu-1* mutants had elevated ROS. Using the ROS-sensitive fluorescent dye 2’,7’-dichlorodihydrofluorescein diacetate (H_2_DCFDA), the accumulation of fluorescent signal over time was measured in lysates prepared from the *nfu-1* mutants. The fluorescent signal accumulated fastest and most significantly in the Gly147Arg mutant and was closely followed by the Gly148Arg and Cys168Phe mutants (Fig 4B and 4B’). Neither the Arg140Gln nor Gly166Cys mutants were significantly different from WT. These data suggested that SKN-1 and/or DAF-16 could be activated by oxidative stress in the *nfu-1* mutants. To further explore this possibility, RNAi was used to determine if *nfu-1* knockdown was sufficient to induce translocation of SKN-1 and DAF-16 to the nucleus in respective GFP-tagged reporter strains (*skn-1b/c::GFP* [53] and *daf-16p*::*daf-16::GFP* [36]). Knockdown of *nfu-1* resulted in nuclear localization of a *skn-1b/c::GFP* transgenic reporter to the same extent as knockdown of *gsk-3*, the negative regulator of SKN-1 (S4B Fig). Knockdown of *nfu-1* also resulted in moderate nuclear localization of a *daf-16p*::*daf-16*::*GFP* transgenic reporter (S4C Fig) although not to the same extent as knockdown of *akt-1*, the negative regulator of DAF-16. Whereas knockdown of *akt-1* resulted in strong nuclear GFP in 100% of animals, only ~75% of *nfu-1* RNAi animals showed mild to moderate nuclear localization of the GFP. Given that *nfu-1* knockdown is less severe than most of the *nfu-1* mutants (S2 Fig), these RNAi data indicated that both SKN-1 and DAF-16 are likely activated in the *nfu-1* mutants.

The glutathione S-transferase gene *gst-4* is a direct target of SKN-1 and a key component of the glutathione system of ROS detoxification. A *Pgst-4::gst-4::GFP* transgene [60] was crossed into the *nfu-1* mutant strains to qualitatively determine the degree of SKN-1 activation and also determine in which tissues SKN-1 might be activated. As expected, the control (transgene only) animals and Arg140Gln mutants had no detectable expression of the transgene (Fig 4C). All other *nfu-1* mutants showed varying degrees of transgene expression in the body wall muscle (Fig 4C). The GFP signal was highest in the Gly166Cys mutant and comparatively low, but detectable, in the Gly147Arg mutant. Surprisingly, obvious GFP expression was not detected in any tissue other than the body wall muscle. It is unlikely that this transgene is expressed in the germline as transgene silencing is very common in this tissue in *C*. *elegans*. However, the lack of expression in the hypodermis, the tissue in which multiple mitochondrial defects had been observed (Fig 3C and 3D), likely indicates that SKN-1 activation was not occurring in that tissue. Notably, expression of the *Pgst-4::gst-4::GFP* does not perfectly correlate with the fluorescence-based ROS measurement (Fig 4B). This finding could indicate a discrepancy between ROS abundance and SKN-1 activation depending on the specific *nfu-1* mutation. It also leaves open the possibility that the oxidative stress response was predominantly mediated by DAF-16 rather than SKN-1.

The H_2_DCFDA and RNAi data suggested that DAF-16 could have a significant role in the detoxification process in the *nfu-1* mutants. Although ATFS-1, SKN-1, and DAF-16 are all capable of increasing expression of detoxifying enzymes, DAF-16 has a distinctive effect on genes such as superoxide dismutases (*sod*) and catalases (*ctl*) which detoxify the superoxide radical and H_2_O_2_, respectively [61]. Thus, expression of these genes was analyzed in the *nfu-1* mutants via qRT-PCR. There was subtle but significant up-regulation of *sod-1* and *sod-2* mRNA in the Gly147Arg mutant with a slight up-regulation of *sod-*2 in the Gly166Cys mutant as well (Fig 4D). However, there was a dramatic up-regulation of *sod-3* in the Gly148Arg and Cys168Phe mutants (Fig 4D). The Gly147Arg and Gly166Cys mutants also appear to have dramatically up-regulated *sod-3*, but these differences were not statistically significant. However, we argue that mean up-regulation of ~45-fold over WT is biologically significant for both mutants. Notably, DAF-16 is known to positively regulate *sod-3* [37,62–64]. Furthermore, the primary *C*. *elegans* catalase genes *ctl-1* and *ctl-2* were up-regulated in most *nfu-1* mutants (Fig 4E). All but the Arg140Gln mutant have up-regulated *ctl-1* whereas only the Gly148Arg and Cys168Phe mutants have significantly up-regulated *ctl-2*. Similar to the circumstance with *sod-3*, the remaining mutants have ~10-fold up-regulation of *ctl-2* although these differences were not statistically significant. Collectively, these data indicate that ROS detoxification pathways are activated, albeit to differing degrees, as a consequence of all *nfu-1* mutations with the exception of Arg140Gln.

The gene expression data presented above are consistent in magnitude and pattern with up-regulation by DAF-16 [61]. To compare the *nfu-1* mutant results with DAF-16 activation, *sod* and *ctl* gene expression were measured in a *daf-2(e1370)* mutant, which has constitutively active DAF-16 at 25°C and no DAF-16 activity 15°C [65]. Consistent with data in the literature, the *daf-2(e1370)* samples raised at 25°C had up-regulated *sod-1*, *sod-3*, *ctl-1* and *ctl-2* (Fig 4D’, and 4E’). Notably, the magnitude of up-regulation of *sod-3*, *ctl-1* and *ctl-2* are consistent with the up-regulation observed in the *nfu-1* Gly148Arg and Cys168Phe mutants (Fig 4D and 4E) suggesting that DAF-16 is responsible for the observed changes in those *nfu-1* mutants. The lower magnitude of change in the Gly147Arg and Gly166Cys mutants could be reflective of incomplete activation of DAF-16. These data thus strongly suggested that DAF-16 has a substantial role in the detoxification response in *nfu-1* mutants with a distinctive effect on mitochondrial ROS detoxification.

Activation of mitochondrial stress responses can result in protection from exogenous mitochondrial stress via mitohormesis, a phenomenon in which the stress responses protect animals from further oxidative stress and contribute to improved fitness [66–68]. To test this possibility in the *nfu-1* mutants, they were exposed to exogenous H_2_O_2_ and paraquat (Pq), a drug that induces production of superoxide. Strikingly, the Gly166Cys mutant was almost completely protected from H_2_O_2_ exposure and the Gly148Arg and Cys168Phe mutants were partially protected from H_2_O_2_ exposure (Fig 4F and Table 2). Surprisingly, the Arg140Gln and Gly147Arg mutants, which were not protected from H_2_O_2_ exposure, were protected from superoxide (induced by Pq exposure) exposure (Fig 4G and Table 2). Neither the Gly148Arg, Gly166Cys nor Cys168Phe mutants were protected from superoxide exposure (Fig 4G and Table 2). Notably, the Gly147Arg and Gly166Cys mutants had nearly opposing protections from exogenous oxidative stress depending on the source of the stress. These data illustrate that the individual *nfu-1* mutations have dramatically different effects on mitohormesis and protection from exogenous stress, potentially indicating meaningful functional differences between the mutants.

**Table 2:** Oxidative stress resistance in *nfu-1* mutants. Median survival and curve difference from WT for both H_2_O_2_ and Pq exposure assays (Fig 4F and 4G). P-values calculated with Log-rank (Mantle-Cox) analysis with Bonferroni correction for multiple comparisons. ND: Not determined.

	H_2_O_2_		Paraquat	
	Median Survival (minutes)	p-value (from WT)	Median Survival (hours)	p-value (from WT)
WT	30	NA	4	NA
Arg140Gln	30	0.9911	4	6.63E-09
Gly147Arg	30	0.8515	5	3.15E-14
Gly148Arg	50	9.33E-05	4	0.712
Gly166Cys	ND	1.67E-24	4	0.1242
Cys168Phe	40	0.1408	4	0.8526

### Disrupted iron homeostasis in *nfu-1* mutants

Due to the fact that ROS measurements, stress responses, and mitohormetic effects were all somewhat inconsistent, the source of ROS in the *nfu-1* mutants was next questioned. Two primary sources seemed apparent: 1) ROS produced by dysfunction of NFU-1 targets; and 2) ROS generated by mishandling of iron in the process of ISC biogenesis. In the second possibility there are two subdivisions: 2a) ROS generated from aberrantly exposed ISCs; and 2b) ROS generated due to reactivity of accumulated mitochondrial iron. In the first possibility, ETC dysfunction is the obvious candidate for ROS generation. CI, CII, and CIII of the ETC naturally produce ROS as a consequence of their respective catalytic activity [69], and CI and CII are known to increase production of ROS when those complexes are dysfunctional [69,70]. As both CI and CII receive ISCs from NFU1/NFU-1, they are likely sources of ROS but ones which were not probed directly.

**Fig 5:**
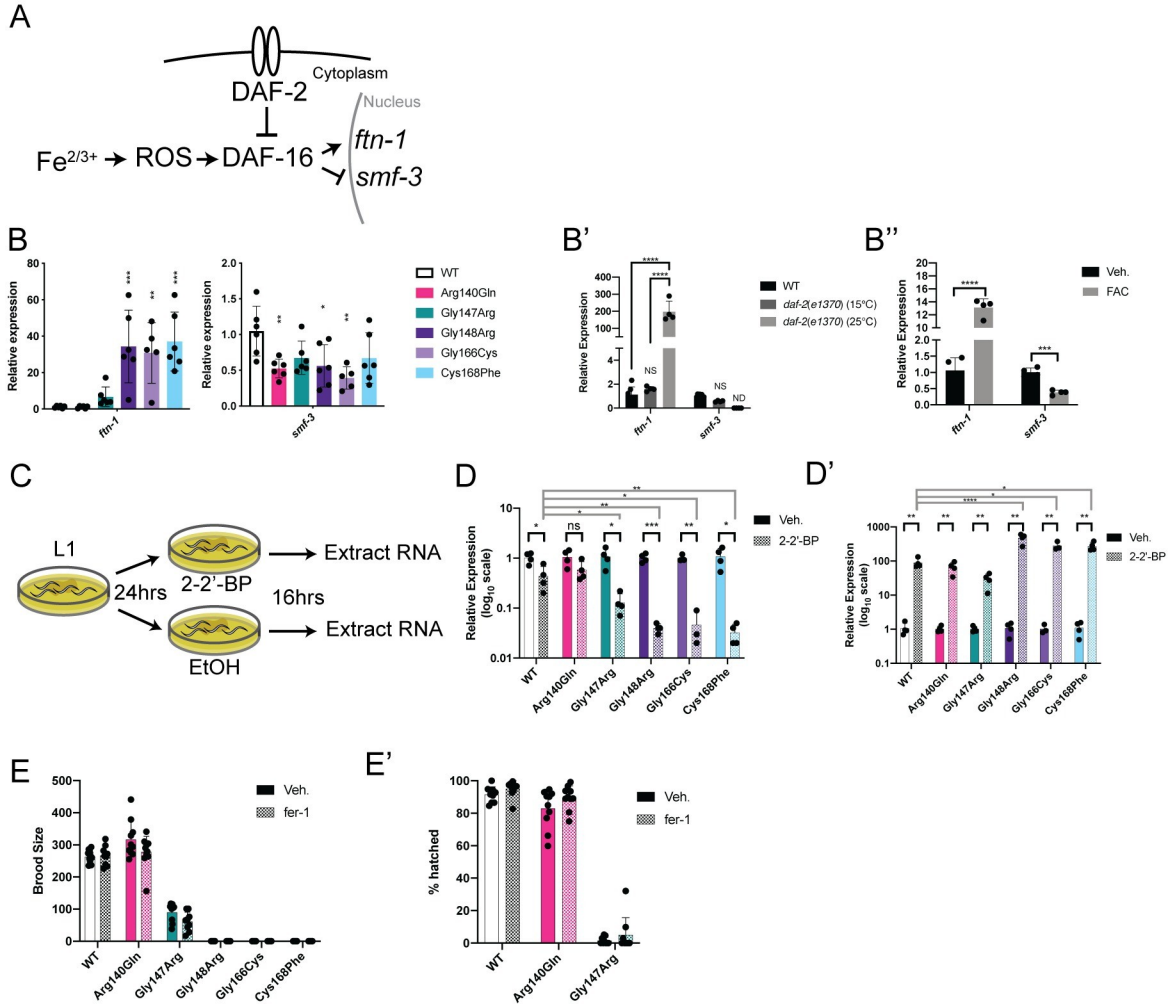
DAF-16-mediated iron response activation in *nfu-1* mutants. (A) Schematic of the DAF-16-mediated iron response pathway. DAF-16 is normally inhibited (indirectly) by DAF-2. Upon disinhibition, DAF-16 translocates to the nucleus where it can activate its transcriptional program including the *C*. *elegans* iron response. (B-B”) Gene expression of *ftn-*1 and *smf-3* (n = 3–6). (B) WT and *nfu-1* mutants; (B’) WT and *daf-2(e1370)* at 15°C or 25°C; (B”) Veh. (H_2_O) or FAC treatment. (C): Schematic of 2–2’-BP treatment. (D-D’) Gene expression of *ftn-1* and *smf-3* following treatment with Veh. (EtOH) or 2–2’-BP (n = 3–5). (D) Expression of *ftn-1*; (D’) Expression of *smf-3*. (E-E’) Fecundity assays following treatment with Veh. (DMSO) or fer-1 (n = 9–10). (E) Total brood size. (E’) Embryonic viability. B-B”: *: p≤0.05; **:p≤0.01; ***:p≤0.001; ****:p≤0.0001 difference between (B) WT and *nfu-1* mutants by One-way ANOVA with Dunnett correction for multiple comparisons; (B’) all groups by One-way ANOVA with Tukey correction for multiple comparisons; or (B”) Vehicle and FAC by parallel two-tailed Student’s T-test with Holm-Sidak correction for multiple comparisons. D-D’ black bars: *: p≤0.05; **:p≤0.01; ***:p≤0.001 between Veh. and 2–2’-BP by parallel two-tailed Student’s T-test with Holm-Sidak correction for multiple comparisons. D-D’ gray bars: *: p≤0.05; **:p≤0.01; ****:p≤0.0001 between fold change of WT and fold change of *nfu-1* mutants as post-hoc One-way ANOVA with Dunnet correction for multiple comparisons. ROS: reactive oxygen species;: Veh.: Vehicle; FAC: ferric ammonium citrate; 2–2’-BP: 2–2’-bipyridyl; ND: Not detected; NS: Not significant.

The second primary possible source of ROS in *nfu-1* mutants is reactive iron, whether in an ISC (2a above) or labile within mitochondria (2b above). Either of these possibilities rely on the reaction of ferrous (Fe^2+^) or ferric (Fe^3+^) iron with H_2_O_2_ to produce a hydroxyl or hydroperoxyl radical, respectively, in a process known as Fenton chemistry (Fig 5A) [71,72]. While it is difficult to directly examine ISC exposure as described in 2a above, iron accumulation has been observed in *nfu1ΔisuΔ* double mutant yeast [39] lending support to the idea that the *nfu-1* mutants could have elevated mitochondrial iron. We attempted to directly assess cellular iron levels with calcein-am, an iron-sensitive fluorescent dye, but were unsuccessful in these attempts. Thus, indirect assessments to infer disrupted iron homeostasis were used.

In mammals, dedicated iron response proteins (IRPs) respond to elevated cellular iron by decreasing iron import through the DMT1 iron transporter and increasing levels of ferritin which sequesters free iron in nanocages [38]. *C*. *elegans* does not have dedicated IRPs, however, they do have an otherwise conserved iron response pathway that is positively regulated by DAF-16 to respond to excessive iron (reviewed in [38]) (Fig 5A). We have already shown that DAF-16 is modestly activated by *nfu-1* RNAi (S4B Fig) and multiple DAF-16 detoxification targets are up-regulated in *nfu-1* mutants (*sod-3*, *ctl-1*, *ctl-2*; Fig 4D and 4E). In direct support of a DAF-16-mediated iron response, it was found that the *C*. *elegans* genes *ftn-1* (orthologous to mammalian *ferritin*) was up-regulated in the Gly148Arg, Gly166Cys and Cys168Phe mutants (Fig 5B). Although less consistent, *smf-3* (orthologous to mammalian *DMT1*) was down-regulated in the Arg140Gln, Gly148Arg, and Gly166Cys mutants (Fig 5B). This response to elevated iron is known to be dependent on DAF-16 [73,74] and these data, as before, are consistent with DAF-16 activation in the *daf-2*(*e1370*) mutant (Fig 5B’). As an additional control, WT animals were loaded with iron by adding 5mM ferric ammonium citrate (FAC) to growth medium and gene expression of *ftn-1* and *smf-3* was measured in L4 animals. As expected, *ftn-1* was increased and *smf-3* was decreased in the FAC-treated samples (Fig 5B”). Intriguingly, the magnitude of the *ftn-1* increase upon FAC treatment is only ~50% of that observed in the *nfu-1* mutants. This difference is possibly a technical limitation of FAC loading or could be indicative of an exacerbating factor in the *nfu-1* mutants. This discrepancy is also consistent with the finding that FAC loading had little to no effect on *sod* or *ctl* expression (S5A and S5B Fig). In fact, *sod-3* expression decreased upon FAC loading potentially indicating that FAC loading affects iron levels within the cytosol more than in mitochondria. Regardless, these results suggest that reactive iron is increased as a consequence of the Gly148Arg, Gly166Cys, and Cys168Phe mutations.

If mitochondrial iron was increased, it seemed possible to suppress the *C*. *elegans* iron response by chelating iron. We chose to chelate iron with 2–2’-bipyridyl (2–2’-BP), a drug that has been used in multiple studies to chelate iron in *C*. *elegans* [73,75–77]. Gene expression analysis following 16-hour exposure to 2–2’-BP (Fig 5C) resulted in the expected decrease in *ftn-1* mRNA and increase in *smf-3* mRNA in all strains (Fig 5D and 5D’). However, the relative decrease in *ftn-1* and increase in *smf-3* were most significant in the Gly148Arg, Gly166Cys, and Cys168Phe mutants (Fig 5D and 5D’). These data, paired with the basal elevation in *ftn*-1 expression in these mutants, strongly suggest that those *nfu-1* mutants have elevated mitochondrial iron. Although not as dramatic as in Gly148Arg, Gly166Cys, and Cys168Phe mutants, the Gly147Arg mutant did have a greater relative reduction in *ftn-1* expression than WT (Fig 5D).

Given the evidence that mitochondrial iron was elevated in the *nfu-1* mutants, the possibility that sterility of the *nfu-1* mutants could be due to ferroptosis was explored. Ferroptosis is an iron-specific form of apoptosis [78–80] that is known to cause sterility in *C*. *elegans* [79,80]. To determine if ferroptosis contributed to sterility of the *nfu-1* mutants, the ferroptosis inhibitor fer-1 was added to the bacterial food source and total brood size was assessed. However, addition of fer-1 had no effect on brood size or embryonic viability of any of the *nfu-1* mutants (Fig 5E and 5E’) nor the delayed egg laying of the Gly147Arg mutant (S5C Fig). Thus, although indirect evidence suggests that cellular iron is elevated in most of the *nfu-1* mutants, ferroptosis alone does not appear to be the cause of sterility in these animals.

### Partial rescue of *nfu-1* mutant lifespan by antioxidants

**Fig 6:**
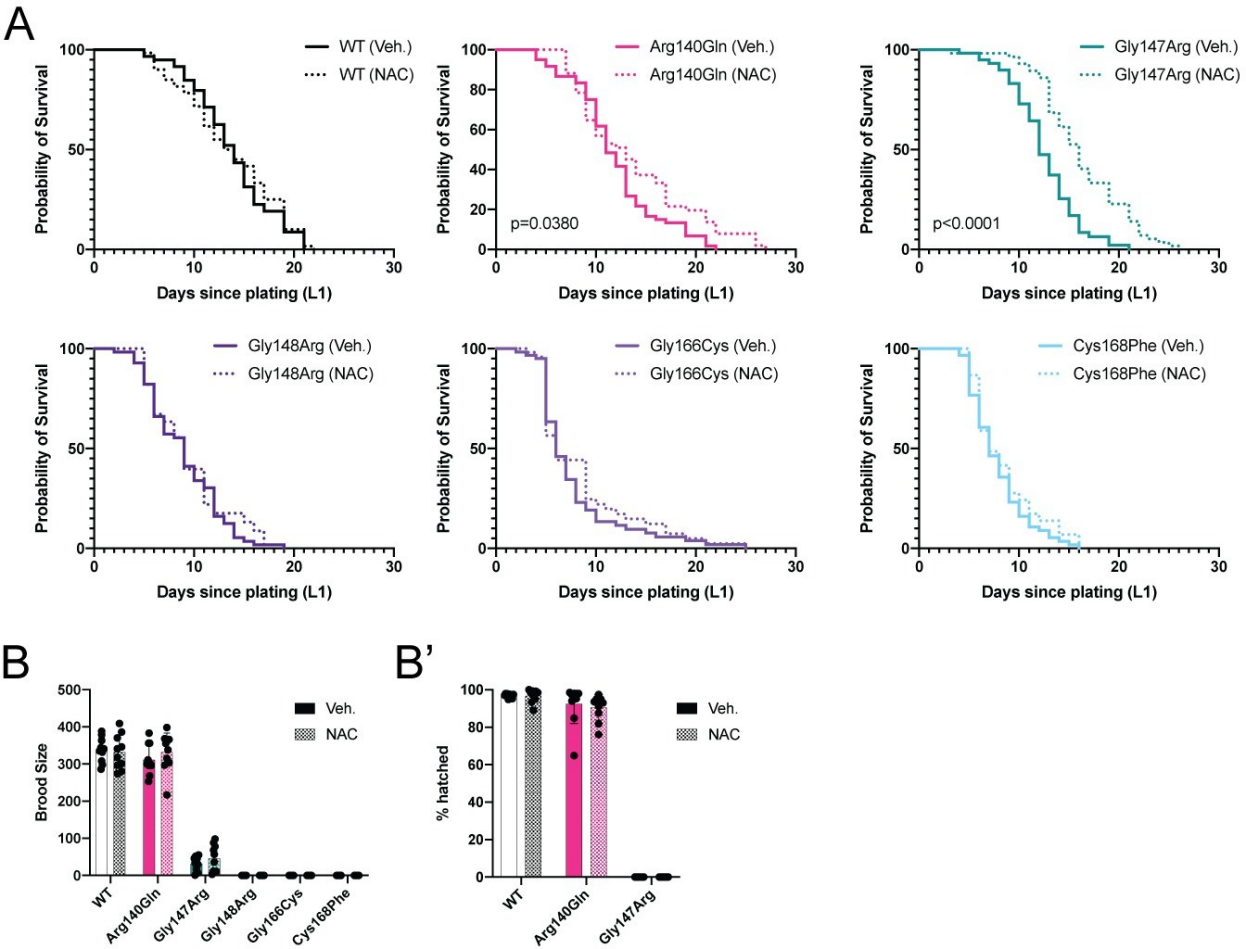
Antioxidant treatment partially suppresses the *nfu-1* phenotype. (A) Kaplan-Meier survival curve of *nfu-1* mutants with either Veh. (H_2_O) or NAC treatment (n = 58–60). Detailed statistical analysis presented in Table 3. (B-B”) Fecundity assays following treatment with Veh. (H_2_O) or NAC (n = 9–10). (B) Total brood size. (B’) Embryonic viability. No statistical differences between treated and untreated. B-B’: Analysis by parallel two-tailed Student’s T-test with Holm-Sidak correction for multiple comparisons. Veh.: Vehicle; NAC: N-acetyl-L-cysteine.

Although inhibition of ferroptosis did not improve sterility of the *nfu-1* mutants, we questioned whether a broad amelioration of oxidative stress could improve lifespan or sterility. The *nfu-1* mutants were treated with 5mM N-acetyl-L-cysteine (NAC), a broadly effective antioxidant, by adding it to the growth medium [70,81]. NAC treatment had no effect on the lifespan of WT animals nor the Gly148Arg, Gly166Cys or Cys168Phe mutants (Fig 6A and Table 3). However, NAC treatment modestly extended the lifespan of the Arg140Gln mutant and significantly extended the lifespan of the Gly147Arg mutant (Fig 6A and Table 3) indicating that oxidative stress is at least a partial cause of the phenotypes observed in the Gly147Arg mutant. Intriguingly, the Gly148Arg, Gly166Cys and Cys168Phe mutants on plates containing NAC presented with a chemoavoidance phenotype that had not been previously observed. These animals were more likely to crawl up the side of the NAC-containing plates and thereby die from desiccation (Table 3, “Suicides”). These animals were censored from our analysis (see Materials and Methods) and we do not have a satisfactory explanation for this phenotype at this time.

**Table 3:** Statistical analysis of NAC treatment lifespans. Median survival (days) and p-value of curve difference as calculated by log rank (Mantle-Cox) analysis for each mutant strain with either Veh. (H_2_O) or NAC treatment. % suicides calculated as number of animals that left the plate divided by the total number of animals assayed. NAC: N-acetyl-L-cysteine.

	Median Survival (days)		p-value of curve difference	% suicides	
Genotype	Veh.	NAC		Veh.	NAC
WT	14	14	0.6932	1.7	0
Arg140Gln	11	13	0.038	0	14
Gly147Arg	12	16	<0.0001	1.7	0
Gly148Arg	9	9	0.5424	3.4	56
Gly166Cys	6	6	0.408	8.3	27
Cys168Phe	7	7	0.2879	6.5	53

Since NAC treatment was sufficient to extend the lifespan of the Arg140Gln and Gly147Arg mutants, the ability of NAC to improve fecundity was tested. Total brood size and embryonic viability was assessed in animals exposed to NAC or vehicle. There was no improvement in brood size or embryonic viability of any *nfu-1* mutant on NAC (Fig 6B and 6B’). Likewise, the delayed egg laying of the Gly147Arg mutant was not improved (S6 Fig). These data demonstrate that suppression of oxidative stress is sufficient to extend the lifespan on the Arg140Gln and Gly147Arg mutants, but not fecundity. Furthermore, suppression of oxidative stress is insufficient to improve the lifespan or fecundity of the Gly148Arg, Gly166Cys, or Cys168Phe mutants, suggesting that multiple factors are likely to contribute to the complex phenotypes of those mutants.

## Discussion

Although only 37 cases of MMDS1 have been reported to date, it is a fatal pediatric disease and one that merits better understanding. Half of MMDS1 individuals are compound heterozygotes for different *NFU1* alleles, thus confounding interpretation of the effects of individual variants. Here, we have used *C*. *elegans* to model patient-specific NFU1 variants and discerned significant differences between them. By analyzing these variants as homozygotes in an otherwise isogenic background, we have been able to differentiate unique characteristics of each variant. Indeed, there are meaningful differences in mitochondrial homeostasis, oxidative stress, and possibly iron homeostasis all arising from the individual variants modeled in *nfu-1*. These differences between the variants determined herein could be significant for their contribution to MMDS1 presentation and pathology as well as the potential for therapeutic intervention. Therefore, this study has sought to elucidate the potential contributions of each allele individually and provide a foundation for future mechanistic and therapeutic studies. All results herein are consolidated in S1 Table.

### NFU-1 mutations and impaired ISC handling

The *nfu-1* mutants generated for this study generally presented as an allelic series with those mutations closest to the canonical CXXC motif most severely affected. The noted exception is the Arg140Gln mutation which is phenotypically normal in almost every assay that was performed. The analogous NFU1 variant (Arg182Gln) is present in three MMDS1 individuals who are all homozygous for this mutation [15]. A similar variant, NFU1 Arg182Thr, has been reported in a single MMDS1 individual who was heterozygous for this variant [19]. Although the NFU1 Arg182 (or NFU-1 Arg140) residue is physically close to the CXXC motif, the human variants have been demonstrated to disrupt a splice donor site in the *NFU1* mRNA rather than ISC handling directly [15,21]. The orthologous mutation is unlikely to impact *nfu-1* mRNA splicing as the mutated nucleotides are not near splice donor or acceptor sites in the *C*. *elegans* gene. As such, it is not surprising that the Arg140Gln mutant does not present with severe phenotypes in *C*. *elegans*. Still, we did observe some subtle phenotypes in this mutant indicating a slight perturbation in NFU-1 function.

**Fig 7:**
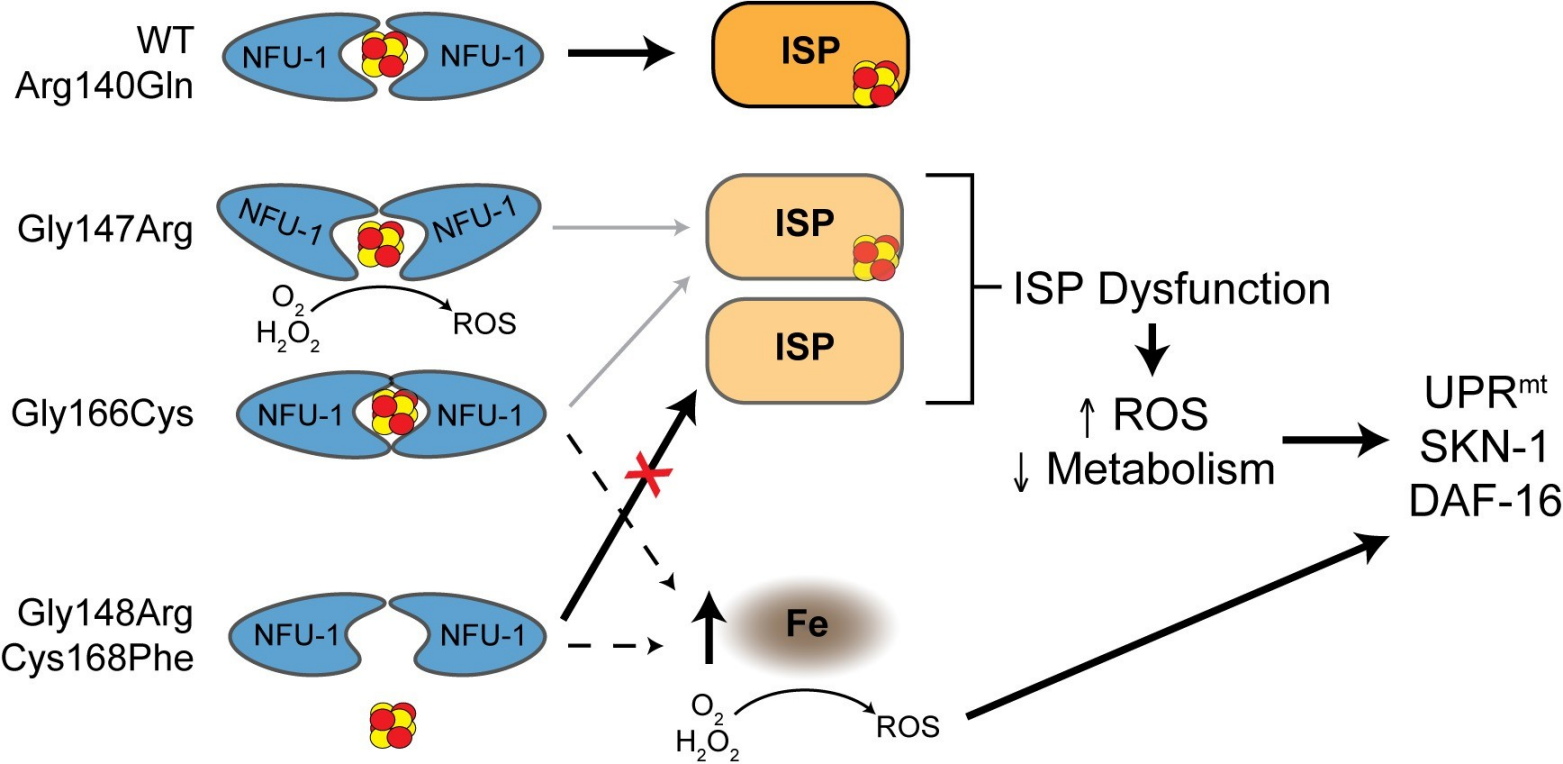
Proposed model for ISC handling and phenotypes of *nfu-1* mutants. Proposed model for how *nfu-1* mutants affect ISC handling and ISC delivery. Model assumes NFU-1 protein functioning as a homodimer. Gly147Arg variant is biased toward monomeric state impairing ISC delivery to ISPs and potentially exposing ISC to reactive environment and producing ROS. Gly166Cys variant is biased towards the dimeric state impairing ISC release and delivery to ISPs. Increased dimerization indicated by NFU-1 proteins more closely surrounding the ISC. Gly148Arg and Cys168Phe are nonfunctional and do not bind ISCs eliminating ISC delivery to ISPs. Gly148Arg, Gly166Cys and Cys168Phe accumulate mitochondrial iron which is reactive via Fenton Chemistry to produce ROS. ISP dysfunction results because of impaired ISC delivery, which leads to elevated ROS and decreased metabolism which in turn activate mitochondrial stress responses. ISP: Iron-Sulfur protein; ISC: Iron-Sulfur cluster; ROS: reactive oxygen species; Fe: labile iron; UPR^mt^: mitochondrial unfolded protein response.

The remaining patient-specific variants modeled herein result in amino acid changes likely to disrupt the normal interaction of NFU-1 with an ISC. However, the nature of that disruption can be dramatically different. *In vitro* data from James Cowan’s group (The Ohio State University) has shown that at least two of these variations affect NFU1 dimerization rather than ISC binding itself. They have performed detailed analyses of both the NFU1 Gly189Arg (NFU-1 Gly147Arg) and Gly208Cys (NFU-1 Gly166Cys) variants and found that the Gly189Arg variant biases NFU1 toward a monomeric state whereas the Gly208Cys variation biases NFU1 toward dimerization, preventing release of ISCs to the recipient ISPs (Fig 7) [82–84]. Therefore, ISC binding and release are affected in both the Gly147Arg and Gly166Cys mutants, but it is a secondary effect to NFU-1 dimerization. The stark difference in dimerization could explain the dramatic differences observed between these two *nfu-1* mutants.

We initially expected the Gly147Arg and Gly148Arg mutants to have similar phenotypes, but this was rarely the case. Rather, the Gly148Arg and Cys168Phe mutants were frequently very similar. As the Cys168Phe mutation abolishes the CXXC motif necessary for ISC handling, this mutant is functionally null, a conclusion supported by comparison with the *nfu-1Δ*. Therefore, the Gly148Arg mutation also appears to result in a fully nonfunctional NFU-1 protein and these two mutants, unlike the Gly147Arg and Gly166Cys mutants, are likely to have phenotypes directly caused by impaired ISC binding. The nuanced differences between these alleles are explored in more detail below.

It is worth noting that some of the phenotypic differences between these mutants are unlikely to be due exclusively to impaired protein lipoylation. The Gly148Arg, Gly166Cys, Cys168Phe and *nfu-1Δ* mutants all lack evidence of lipoylated proteins (Fig 1E). Yet, the phenotypic presentation of these mutants is not always identical, especially with the Gly166Cys mutant, indicating auxiliary stresses or dysfunctions. Recent work with a rodent model of the human NFU1 Gly208Cys mutant has also shown similarly complex phenotypes to the orthologous *C*. *elegans* Gly166Cys mutant including decreased ETC function, oxidative stress, and mitohormesis [85]. While some of these findings are unique to the Gly166Cys mutant, many are shared with the Gly148Arg and Cys168Phe mutants as well, further illustrating complexity beyond reduced protein lipoylation. Finally, the Gly147Arg mutant frequently presented with unique phenotypes. This mutant also had partially impaired, but not abolished, protein lipoylation. It is unclear at this time what the effects of impaired lipoylation are in comparison to abolished protein lipoylation since the Gly147Arg mutant appears to be affected by confounding stressors. Two final considerations must be made when interpreting these data. First, we were unable to determine whether or not NFU-1 protein levels are changed in the *nfu-1* mutants. Data from MMDS1 patient-derived fibroblasts is inconclusive but seems to indicate that only the human Arg182 mutations result in severe mRNA and/or protein degradation [15,16,21,28]. This information, paired with the data herein about diffences between alleles, suggests that the amino acid changes are likely the causes of dysfunction rather than reduced NFU-1 protein abundance. Second, recent work has demonstrated that the C-terminus of NFU1, including residues surrounding the ISC-binding motif is important for interactions with both other members of the ISC biogenesis machinery and NFU1 targets [9,11]. We cannot exclude the possibility that the modeled *nfu-1* mutants also disrupt those types of interactions and likewise alter ISC handling.

### Effects of ETC dysfunction in *nfu-1* mutants

The Seahorse data clearly demonstrated that all *nfu-1* mutants have a dysfunctional ETC with the Gly148Arg, Gly166Cys, Cys168Phe and *nfu-1Δ* mutants being the most severe (Fig 3A and 3A’). The likeliest explanation for this finding is that the failure of these mutants to effectively deliver ISCs to CI and CII [12,13,70] precludes normal ETC function. This conclusion is in agreement with the recent rodent model of MMDS1 [41,85]. Given that the ETC is responsible for maintaining Δψ^m^, the TMRE data supports this conclusion (Fig 3D and 3E). However, there were intriguing observations with the Arg140Gln and Gly147Arg mutants. Despite only a slight difference in maximal OCR, the Arg140Gln mutant had elevated TMRE labeling. Although speculative, this finding could suggest that slightly elevated ETC activity (below detection of the Seahorse) further hyperpolarizes Δψ^m^. Such an increase in ETC activity could be necessary if an otherwise undetected impairment in function were to exist. The more striking finding is that the Gly147Arg mutant also has only a modest reduction in maximal OCR (Fig 3A and 3A’) but dramatically decreased TMRE labeling (Fig 3D). This finding demonstrates that a factor other than ETC dysfunction is the cause of the reduced Δψ^m^. As will be discussed below, the likeliest cause is ROS.

ETC dysfunction is also likely a significant contributor to the two most obvious phenotypes of the *nfu-1* mutants: sterility and developmental delay/arrest. The arrest was variable between all mutants and most severe in the Gly148Arg, Gly166Cys and Cys168Phe mutants, which arrested at an early L4 stage. It was for that reason that all assays herein were carried out with L4 larvae. Developmental arrest and sterility are common among mitochondrial mutants [43,86]. While the exact mechanism leading to sterility is unclear, it is thought that reduced metabolic capacity precludes the development of germ cells which have a high ATP requirement [87,88]. Thus, ETC dysfunction in the *nfu-1* mutants is likely a determining factor in germ cell apoptosis. The fact that neither inhibition of ferroptosis nor supplementation with NAC improved fecundity of the *nfu-1* mutants supports this hypothesis. Yet, the question remains as to why *nfu-1* mutants arrest slightly later than other mitochondrial mutants. Our RNAi data suggests that a maternal contribution of the NFU-1 protein is diminished at approximately the late L3 or early L4 stage (S2 Fig). This coincides with the arrest of the severely affected *nfu-1* mutants. As such, it seems likely that this maternal rescue precludes development of severe phenotypes prior to the L4 stage.

### Oxidative stress and iron dysregulation

Multiple lines of evidence presented herein indicate that oxidative stress contributes to the phenotypes observed in the *nfu-1* mutants as well as the corroborating evidence in a rodent MMDS1 model [85]. The most direct of these is the elevated fluorescence observed in the Gly147Arg, Gly148Arg and Cys168Phe mutants in the H_2_DCFDA assay (Fig 4B and 4B’). This, paired with the dramatic up-regulation of antioxidants (Fig 4C, 4D, and 4E), strongly suggests that many tissues are experiencing oxidative stress. As alluded to in the previous section, CI and CII dysfunction caused by reduced or absent ISCs generates ROS [69,70]. The most dramatic change in gene expression was up-regulation of *sod-3*, the mitochondrial-specific superoxide dismutase, which would be necessary to neutralize superoxide radicals produced by a dysfunctional CII [70]. Thus, it seems very likely that ROS generated by the ETC are a contributing factor to the observed oxidative stress.

Another possible source of ROS is reactive iron that could exist in one of two forms in the *nfu-1* mutants. The first is labile mitochondrial iron that accumulates as a result of impaired ISC biogenesis and, more specifically, delivery. In such a model, the failure to use mitochondrial iron for the biogenesis of ISCs results in a back up of the ISC biogenesis machinery and iron to accumulate prior to utilization. Mutations to frataxin, another member of the ISC biogenesis pathway, are known to cause elevated mitochondrial iron in yeast and mammals (reviewed in [89]). Likewise, loss of function of the ISP CISD3/MiNT also results in labile iron accumulating in mitochondria [90]. Unfortunately, we were unsuccessful in attempts to directly assess mitochondrial iron content in the *nfu-1* mutants, but the data support a mitochondrial iron-dependent response in the Gly148Arg, Gly166Cys and Cys168Phe mutants (Fig 5). The data are less consistent for the Gly147Arg mutant potentially because it retains some ISC delivery function as indicated by the incomplete, but existent, lipoylation of proteins (Fig 1E). That the Gly148Arg and Cys168Phe mutants are functionally null suggests that elevated mitochondrial iron rather than mishandling of an ISC is the source of reactive iron in those mutants (Fig 7). Such a finding is in agreement with the frataxin examples referenced above [89].

Perhaps the most intriguing source of ROS is from the ISCs that are bound by NFU-1. The likeliest example of this paradigm is in the Gly147Arg mutant. NFU1/NFU-1 coordinates ISCs as a dimer, and it is believed to typically function as a homodimer although other binding partners are known [8]. Assuming that NFU-1 is functioning as a homodimer, the Gly147Arg mutant is more likely to expose an ISC to the environment because of the fact that this mutation biases NFU1/NFU-1 to the monomeric state [82,84]. If the ISC coordinated by this mutant is exposed to the environment, it can react with H_2_O_2_ via Fenton chemistry or with O_2_ via redox mechanisms. This could explain why this mutant appears to have the highest ROS burden (Fig 4B and 4B’) and why it has elevated *sod-1* expression and is protected from exogenous Pq exposure. Finally, the fact that this mutant is the only one to significantly benefit from antioxidant treatment further supports the conclusion that the mechanism of oxidative stress is different from that of the other *nfu-1* mutants.

The possibility that iron, whether labile or in an ISC, is reacting with cellular O_2_ is further supported by mounting evidence that hypoxia can suppress mitochondrial dysfunction. Hyperoxia has been shown to abrogate the effects of antioxidants in an SDHB mutant [70] suggesting that elevated O_2_ is in fact detrimental in the context of ETC dysfunction. More recent work has shown that hypoxia is sufficient to suppress not only defects of an *frh-1* mutant, but also over 200 other genes including most genes associated with ISC biogenesis [91,92]. Notably, *NFU1* is included in this list of suppressed genes [91,92]. This suppression is HIF-1 independent lending support to the role of O_2_ as a reactive species in and of itself [92]. However, at this time, it remains unclear how the individual mutations modeled here are affected by cellular O_2_ levels.

### Complex activation of stress response pathways

Multiple pieces of evidence presented herein indicate the activation of ATFS-1, SKN-1, and DAF-16 as regulators of stress responses activated in *nfu-1* mutants. It must be noted that these transcription factors are capable of interacting to affect each others’ activity in certain contexts. For example, ATFS-1 is necessary for full activation of DAF-16 during development in a *nuo-6* mutant [57]. Alternatively, prolonged SKN-1 activation can inhibit DAF-16 activity [93]. Therefore, it remains unclear at this time to what extent these transcription factors and pathways genetically or physiologically interact in the *nfu-1* mutants despite the evidence for DAF-16 mediated stress reponses.

A second complexity to understanding the stress responses in *nfu-1* mutants is the potential cell-nonautonomous activation of these pathways. While much of our data supports metabolic and oxidative stresses acting cell-autonomously to activate response pathways, both SKN-1 and DAF-16 can be activated cell-nonautonomously [32]. That is, a stressor in one cell can trigger the activation of a response factor in a different cell. We have observed circumstantial evidence of this with the nuclear accumulation of *skn-1b/c::GFP* in intestinal nuclei upon *nfu-1* knockdown (S4 Fig), yet expression of the *Pgst-4::gst-4::GFP* reporter is only evident in the body wall muscle of mutant animals (Fig 4C). These assessments of SKN-1 activation differ in many ways, but they demonstrate the point that cell-nonautonomous activation could be possible. Furthermore, in *C*. *elegans*, metabolic dysfunction in neurons has been documented to activate stress responses in other cells, namely the intestine [94,95]. Such a concept is pertinent given the neurodegenerative nature of MMDS1 and the potential for neuronal dysfunction to impact nonneuronal tissues. Nearly all MMDS1 individuals present with neurodegenerative symptoms which are likely to affect, if not be causative of, both the hypotonia and pulmonary hypertension that are also characteristic of MMDS1 [41,85]. Thus *C*. *elegans* could provide an excellent model in which to parse the nuances of these stress-response relationships further.

In conclusion, we find here that patient-specific *NFU1* variants modeled in *nfu-1* result in unique dysfunction and stress in *C*. *elegans*. Although some effects of these mutations are consistent, such as decreased protein lipoylation and decreased ETC function, the compensatory responses differ in appreciable ways. Altered expression of antioxidants and production of exogenous oxidative stress markedly differ between the mutants. Further, the ability of exogenous antioxidants to improve the lifespan of the mutants also widely differs. Collectively, these data lead us to present the model in which the specific mechanisms by which ISCs are mishandled, and the cellular consequences therein, are dictated by the specific mutation to the ISC interaction domain. This work thus illuminates some of the nuanced pathogenicity of the many variations documented in MMDS1.

## Materials and methods

### *C*. *elegans* maintenance

*C*. *elegans* were maintained with *E*.*coli* OP50 at 20°C on MYOB plates. Strains used in this study are reported in Table 4. Sterile mutant *nfu-1* strains were balanced with the *tmC5* balancer chromosome [96]. Synchronized populations were obtained by lysis of gravid adult animals with sodium hypochlorite treatment. Embryos were either plated directly onto MYOB plates or allowed to hatch overnight (at 20°C, rotating) in S-basal medium allowing for the generation of a synchronous population of starved L1 larvae.

**Table 4:** Strains used in this study.

Strain	Genotype	Origin
N2	WT	CGC
AG264	*nfu-1* (*av57*) [Arg140Gln]	This study
AG377	*nfu-1* (*av115*) [Gly147Arg]/*tmC5*	This study
AG428	*nfu-1* (*av159*) [Gly148Arg]/*tmC5*	This study
AG378	*nfu-1* (*av116*) [Cys168Phe]/*tmC5*	This study
AG561	*nfu-1* (*av56*) [Gly166Cys]/*tmC5*	This study
JJ2586	*cox-4* (*zu476*[*cox-4*::*eGFP*::*3xFLAG*])	[49]
AG411	*nfu-1* (*av56*) [Gly166Cys]/*tmC5*; *cox-4*(*zu476*[*cox-4*::*eGFP*::*3xFLAG*])	This study
AG412	*nfu-1* (*av57*) [Arg140Gln]; *cox-4*(*zu476[cox-4*::*eGFP*::*3xFLAG*])	This study
AG413	*nfu-1* (*av116*) [Cys168Phe]/*tmC5*; *cox-4*(*zu476*[*cox-4*::*eGFP*::*3xFLAG*])	This study
AG477	*nfu-1* (*av115*) [Gly147Arg]/*tmC5*; *cox-4*(*zu476*[*cox-4*::*eGFP*::*3xFLAG*])	This study
AG478	*nfu-1* (*av159*) [Gly148Arg]/*tmC5*; *cox-4*(*zu476[cox-4*::*eGFP*::*3xFLAG*])	This study
MJCU017	*kIs17* (*Pgst-4::gst-4::GFP *)	[60]
AG471	*nfu-1* (*av56*) [Gly166Cys]/*tmC5*; *kIs17* (*Pgst-4::gst-4::GFP*)	This study
AG472	*nfu-1* (*av57*) [Arg140Gln]; *kIs17* (*Pgst-4::gst-4::GFP*)	This study
AG473	*nfu-1* (*av115*) [Gly147Arg]/*tmC5*; *kIs17* (*Pgst-4::gst-4::GFP*)	This study
AG474	*nfu-1* (*av116*) [Cys168Phe]/*tmC5*; *kIs17* (*Pgst-4::gst-4::GFP*)	This study
AG475	*nfu-1* (*av159*) [Gly148Arg]/*tmC5*; *kIs17* (*Pgst-4::gst-4::GFP*)	This study
NL2098	*rrf-1* (*pk1417*)	[97]
VP303	*kbIs7* [*nhx-2p*::*rde-1 + rol-6*(*su1006*)]	[98]
AMJ345	*jamSi2* [*mex-5p*::*rde-1(+)*] II	[99]
NR350	*kzIs20* [*hlh-1p*::*rde-1 + sur-5p*::*NLS*::*GFP*]	[100]
LD1	*ldIs7* [*skn-1b/c*::*GFP + rol-6(su1006)*]	[53]
TJ356	*zIs356* [*daf-16p*::*daf-16a/b*::*GFP + rol-6(su1006)*]	[36]
SJ4100	*zcIs13* [*hsp-6p*::*GFP + lin-15(+)*]	[58]
CB1370	*daf-2 (e1370)*	[101]

### CRISPR/Cas9 design and editing

The Bristol N2 strain was used as the wild-type parent strain for all CRISPR/Cas9 editing of *nfu-1*. Site-specific 20-nucleotide guide sequences for crRNA were manually determined and generated by Dharmacon (now Horizon, a PerkinElmer company) who also provided the tracrRNA. Repair template design followed established protocols [102]. Injection mixes were prepared following established protocols [102] and *dpy-10* was used as a co-CRISPR selection marker. Injection mixes for the *nfu-1Δ* allele required two guide RNAs. Guide RNA and repair sequences are reported in Table 5. Correct substitution or deletion sequences were confirmed via Sanger sequencing.

**Table 5:** CRISPR guide RNA and repair sequences. All sequences reported in 5’– 3’ orientation.

Mutant	Guide Sequence	Repair Oligo
Arg140Gln	GACCAATGGTACAAGAA	ACCAAGTGGAAGAAGATGATGAAGTTGTAATGATGATCAAAGAAATTCTAGAGACTCGAATTCAACCGATGGTTCAGGAGGACGGTGGAGATATCACTTATGTTGGTTTTGACGATGGTGTTGTCAAATTGAAGA
Gly147Arg	AAUGGUACAAGAAGAUGG	ATTCTAGAGACACGTATCAGACCAATGGTACAAGAAGACCGTGGCGACATCACTTATGTTGGTTTTGACGATGGTGTTGTCAA
Gly148Arg	AAUGGUACAAGAAGAUGG	ATTCTAGAGACACGTATCAGACCAATGGTACAAGAAGACGGCAGGGACATCACTTATGTTGGTTTTGACGATGGTGTTGTCAA
Gly166Cys	AGATGCAAGGTTCATGCAC	GGTACAAGAAGATGGTGGAGATATCACTTATGTTGGTTTTGACGATGGTGTTGTCAAACTTAAAATGCAATGTTCATGCACAGGATGTCCAAGCTCTGGAGTCACTTTAAAAAATGGAATTGAG
Cys168Phe	GAUGCAAGGUUCAUGCAC	GGTTTTGACGATGGTGTTGTCAAATTGAAGATGCAGGGATCCTTCACTGGACAGGATGTCCAAGCTCTGGAGTCACTTTAAAA
*nfu-1*Δ	CAAUGUAUAAACAAGUUC	TTATACACGAATCAATTATCAATTAGTCTTTGATTTCTTTTTCACTTGTAGAGCTCGTCCA
	GAAAUUUGAGCAAUCAAA	
*dpy-10*	UACCAUAGGCACCACGAG	CACTTGAACTTCAATACGGCAAGATGAGAATGACTGGAAACCGTACCGCATGCGGTGCCTATGGTAGCGGAGCTTCACATGGCTTCAGACCAACAGCCTAT

### Gene expression knockdown via RNA interference

F_1_ animals were analyzed for phenotypes caused by RNAi-mediated knockdown. L4 parental animals (P_0_) were plated on RNAi plates for 24 hours. P_0_ animals were then plated individually onto RNAi plates and allowed to lay embryos for 24 hours prior to removal from the plate. F_1_ progeny of the P_0_ were maintained on RNAi plates and analyzed every 24 hours thereafter for fecundity, developmental progress, viability or other relevant phenotypes.

### Complementation assay

cDNAs for human *NFU1*, and *C*. *elegans nfu-1*/*lpd-8* were derived from total RNA from U206 cells and N2 animals, respectively. *S*. *cerevisiae NFU1* cloned into p416-Met25 [42] was generously provided by Drs. Ulrich Mühlenhoff (Philipps-Universität, Marburg) and Nicolas Rouhier (Universite de Lorraine, Vandoeuvre-Lés-Nancy). Strain YKL040C::KanMX (nfu1D) from the yeast knockout library was transformed with URA3 plasmids (p416-GPD or p416-Met25) encoding *S*. *cerevisiae nfu1*, *H*. *sapiens NFU1*, *C*. *elegans nfu-1/lpd-8* or an empty vector control using the lithium acetate method. Transformants were selected on synthetic complete (Sunrise Scientific) media lacking uracil (SC -Ura) containing 0.67% yeast nitrogen base and 2% dextrose. Solid media contained 2% agar. Transformants were grown overnight in liquid SC -Ura then serially diluted and spotted onto SC -Ura and identical media that contained 2% sodium acetate instead of dextrose. Plates were incubated at 30°C and imaged after 3–5 days.

### Predicted protein structure

NFU-1 protein structure was predicted using the Phyre2 web tools. [103]. Analysis of the predicted structure was carried out with the EZMol interface [104]. NFU-1 structure is predicted and not experimentally derived.

### Western blot

Synchronized populations of 5,000 L4 animals were collected for each biological replicate. For balanced strains, a COPAS (Union Biometrica) biosorter was used to select for homozygous mutants. Samples were washed 5X in M9 prior to lysis in 100μL RIPA buffer (150mM NaCl, 1% NP-40, 0.5% Sodium Deoxycholate, 0.1% SDS, 25mM Tris [pH 7.4]) with 1X phosphatase and 1X protease inhibitors (ThermoFisher), frozen at -80°C and homogenized with a Branson 250 Sonifier operating for 5 cycles at 50% duty cycle and output set to 6. Lysates were pelleted and the supernatant was frozen at -20°C for storage. Protein concentration was determined with a Pierce BCA Protein Assay Kit (Thermo Scientific). 15μg protein was loaded per well with NuPage LDS loading buffer (ThermoFisher) and 4mM DTT, run on a Novex 4–12% Bis-Tris gel (ThermoFisher) before transfer to nitrocellulose membrane (ThermoFisher). Membranes were blocked for 1 hour with Odyssey Blocking Buffer (PBS)(LI-COR) prior to incubation with primary antibodies in PBST (1X PBS + 0.01% Triton X-1000) overnight at 4°C. Primary antibodies were from the Developmental Studies Hybridoma Bank: mouse anti-β-Tubulin (E7; AB_2315513; 1:2,500) and EMD Millipore Rabbit anti-Lipoic Acid (pAB 437695; 1:1,000). After washing 5 X 10’ with PBST, membranes were incubated for 2 hours at room temperature with secondary antibodies (anti-mouse or anti-rabbit conjugated to IRDye680 or IRDye800; LI-COR) diluted in PBST. Membranes were washed 5 X 10’ with PBST and imaged using the Odyssey Imaging System (LI-COR). Ladder used was Chameleon Duo Pre-stained Protein ladder (LI-COR).

### Lifespan and brood size

For lifespan analyses, L1 animals were plated on MYOB plates seeded with OP50. Viability was assessed daily as response to gentle touch with an eyelash pick. If animals were fertile, they were transferred to a new plate daily until the cessation of laying. For brood size analyses, L1 animals were plated individually on MYOB plates seeded with OP50. Animals were transferred to a new plate daily until the cessation of laying. Viability of embryos was assessed 24 hours after removal of the parent animal.

### Drug treatments

Assays performed as described above with the addition of drug or vehicle control. For fer-1, either Vehicle (10% DMSO in H_2_O) or fer-1 (250μM in 10% DMSO; Millipore Sigma #SML0583) was added directly to the OP50 spot and allowed to dry 30’ prior to plating animals. Vehicle or fer-1 was added fresh daily prior to transferring animals to new plates. For NAC, either Vehicle (H_2_O) or NAC (5mM final concentration) was added to MYOB media immediately before pouring into plates. Total volume added was 0.8% (v:v) of total. In the NAC treatment, animals that crawled up the side of the plate and subsequently dehydrated were censored from the analyses since the death was not attributable to the potential oxidant scavenging effect of NAC.

### Fluorescent Imaging

#### Confocal microscopy

Confocal microscopy was carried out using a Nikon Eclipse Ti2 microscope equipped with a Yokagawa CSUX-1 spinning disk and Photometrics Prime95B camera using Nikon NiS Elements acquisition software, a Nikon Eclipse E800 inverted phase contrast microscope equipped with a Hamamatsu ORCA-flash 4.0 LT+ camera using Metamorph acquisition software, or a ZeissAxioObserver.Z1 microscope equipped with a Zeiss LSM 780 confocal scanhead using Zen 2.3 software. Images were captured in widefield at 10X magnification or high magnification at 60X or 63X using an oil immersion or water immersion objective depending on sample preparation.

Live imaging was carried out by washing animals 3-5X in M9 prior to mounting on a 10% agarose pad. Samples were coverslipped, sealed with nail polish, and immediately imaged. Imaging of fixed samples is detailed below. Z-stacks were captured with 0.3μm steps.

#### DAPI staining

L4 animals from a synchronous population were picked from the plate into a watch glass of M9 to remove excess bacteria. Animals were then picked into a drop of 1:1 egg white:M9 solution on a slide. When worms were just dried onto the slide they were immersed into Carnoy’s fixative (10% acetic acid, 30% chloroform, 60% EtOH) and fixed overnight at 4°C. Slides were rehydrated through an EtOH series (100%, 90%, 75%, 50%, 25%) for 2’ each and rinsed 5’ in PBS. Slides were incubated for 10’ in DAPI solution (10μg/mL), rinsed for 5’ in PBS, and mounted with Vectashield (Vector Laboratories). Image analysis including 3D reconstructions of gonads was carried out with Imaris Software (BitPlane). A custom automated protocol was developed to identify and count germline nuclei.

#### TMRE labeling

L4 animals from a synchronous population were washed 3 times with M9 prior to incubation in 200nM TMRE (ThermoFisher) in M9 for 6 hours in the dark. Following incubation, animals were washed 3 times with M9 and mounted on 10% agarose pads for imaging.

#### TUNEL imaging

TUNEL imaging was carried out as in [105] using the TUNEL Assay Kit (Abcam). In brief, gonads were dissected from L4 with a needle prior to fixation in 4% paraformaldehyde (PFA) for 20’ at room temperature. Samples were rinsed 3 times with PTX (1XPBS +0.4% Triton-X1000) for 3’ each, incubated 20’ at 65°C in 100mM Sodium Citrate buffer + 0.1% Triton X-1000, rinsed as above, and incubated for 1 hour at 37°C in the TUNEL reaction mix (following manufacturer’s recommended protocol). Samples were rinsed as above, incubated in 5’ in DAPI solution (10μg/mL), rinsed as above, and mounted with Vectashield (Vector Laboratories).

### Oxygen consumption

Analysis of oxygen consumption rate (OCR) was based on the protocol in [45]. In brief, synchronized populations of L4 animals were rinsed 5 times in M9 to remove bacteria including a 20’ incubation to allow animals to clear their intestines. ~40 animals were added to each well of an xFe96 assay plate and the assay was begun within 30 minutes of plating. Measurements were carried out in an Agilent Seahorse xFe96 bioanalyzer. Measurements were carried out as follows: four measurements for basal OCR, eight measurements for Complex IV-independent OCR, nine measurements for maximal OCR, and four measurements for respiration-independent OCR. Blockage of Complex IV was achieved with dicyclohexylcarbodiimide (DCC; 20μM final concentration), maximal respiration was achieved with Carbonyl cyanide-4-phenylhydrazone (FCCP; 25μM final concentration), and blockage of Complex V was achieved with sodium azide (NaN_3_; 40mM final concentration). Following runs, data were normalized to the number of worms per well. Data were collected from three independent assays with a minimum of three biological replicates per assay.

### Oxidative stress assays

#### H_2_DCFDA fluorescence assay

Synchronized L4 animals were washed from plates, washed three times with M9 and a final wash in PBST (1X PBS + 1% Tween-20). Wet pellets of animals were resuspended in 100μL PBST. Samples were sonicated as described in the Western blot protocol. 50μL of lysate was used for the fluorescence assay. To the 50μL lysate, 50μL H_2_DCFDA solution (20μM in 20% DMSO) was added for a final concentration of 10μM in PBST+10% DMSO. Immediately upon addition of H_2_DCFDA, plate was loaded into a prewarmed (37°C) POLARstar Omega plate reader (BMG Labtech) with fluorescence measurements (excitation: 504nm; emission: 529nm) taken every 10’ for 150’. Fluorescence signal for each sample was normalized to protein concentration as determined by Pierce BCA Protein Assay Kit (Thermo Scientific). Data collected from three independent assays.

#### Exogenous oxidant stressor assays

Sensitivity to exogenous oxidative stress was assayed by either soaking L4 animals in 0.01% H_2_O_2_ or placing animals on plates containing 200mM paraquat. For the H_2_O_2_ stress assay, each well containing ~10 animals was considered a biological replicate. Data was collected from 2 independent assays. For the paraquat stress assay, ~25 animals were plated into each well of a 12-well plate containing MYOB medium supplemented with 200mM paraquat. A small drop of OP50 was included in each well. Data was collected from two independent assays. Animals were considered dead when they no longer responded to touch by an eyelash pick at the head, mid-body, or tail. Data were collected as a percent of the population in each well still alive and moving.

### Analysis of gene expression

#### RNA extraction

RNA extractions were carried out as in [106]. In brief, synchronized populations of L4 animals were washed 5 times with M9, pelleted, resuspended in 500μL TRIzol (ThermoFisher), and frozen at -80°C for lysis. Samples were vortexed for 15’ at 4°C prior to RNA precipitation with isopropanol. RNA was processed with the QIAGEN RNEasy kit or Zymo Quick-RNA miniprep kit following the manufacturers’ recommended protocol. Within any comparison herein, the same kit had been used for RNA processing. Concentration of RNA samples was measured on a Nanodrop Lite (ThermoFisher) and stored at -80°C until cDNA synthesis.

#### cDNA synthesis and quantitative reverse-transcription PCR

cDNA synthesis was carried out using the Invitrogen SuperScript III First-Strand Synthesis System following the manufacturer’s recommended protocol for Oligo dT primers or the BioRad iScript cDNA Synthesis Kit following the manufacturer’s recommended protocol. Within any comparison herein, the same kit had been used for cDNA synthesis.

Quantitative reverse-transcription PCR (qRT-PCR) was carried out in technical replicates with the SYBR Select Master Mix (Applied Biosystems) on either a CFX96 or CFX384 Touch Real-Time PCR Detection System (Bio-Rad). Primers used for this study are listed in Table 6. Analysis of gene expression was performed with CFX Manager software.

**Table 6:** Primers used in this study. Forward and reverse primers indicated. All sequences oriented 5’ to 3’.

Target gene	Forward/Reverse	Sequence (5’-3’)	
*act-1*	For.	CAGAAGGAAATCACCGCTCTT	
	Rev.	ATAGATCCTCCGATCCAGACG	
*nfu-1* [52]	For.	GTCCAACAGCTTCTCCCAGACG	
	Rev.	GCTTCACTCCGTCGACTCG	
*gas-1*	For.	GGAGTTTACCTTGTTGCTGATG	
	Rev.	ACAATGTCGGCGATAAGAGA	
*mev-1*	For.	AGGATTCGATTTGGCTAAGGG	
	Rev.	AGTTGAAGACAATGGCGAGAG	
*clk-1*	For.	TGTGGCTGCTTATGCTCTC	
	Rev.	GTTGTCCAATGAGTTCTTCAACTG	
*isp-1*	For.	GGAGGTTATTACTGCCCTTGT	
	Rev.	GAGTATGCTGGTACGTGAAGAT	
*cox-4*	For.	TTGTCTGCTGAGGAGAAGAAG	
	Rev.	CGGAGATCACCTTCCAGTATC	
*asg-2*	For.	CAAAGTCCAGAGCTTCATCCA	
	Rev.	AGAACCAGAAGACAACCTCAAG	
*fzo-1*	For.	CAATGCGATGCTTCATGAGAAA	
	Rev.	CAACTTCGCCTTCTGAACCT	
*drp-1*	For.	TGATCGTGGATGCAGTGAAAG	
	Rev.	GCAGGAAGACAGTTGCGAATA	
*dct-1*	For.	ACTGATTGGATTTGGGATTGGA	
	Rev.	GAGTTTGGCGGAGTGGTAAG	
*atfs-1* [51]	For.	CCACGCATTCAAGTACAACAG	
	Rev.	GGAAGTTCCCGTTAGCTGAT	
*hsp-6*	For.	CTTTGACATTGACGCTAATGGTATC	
	Rev.	GGAAAGTCCTCCAGAAGATTGG	
*dnj-10*	For.	GCGGGCTCATTCATCGATCTGTAC	
	Rev.	CAGATTTTTTGTCGACACCCAAAG	
*sod-1*	For.	GGTCCACACTTCAATCCATTTG	
	Rev.	CCAGCTTCCACATTTCCTAGAT	
*sod-2*	For.	GCATCATGCCACTTATGTGAAC	
	Rev.	GAACTTGAGAGCTGGCTGAA	
*sod-3*	For.	GGATGGTGGAGAACCTTCAAA	
	Rev.	AGAGCCTTGAACCGCAATAG	
*ctl-1*	For.	TGCTGAACAGGAATGTGAAGA	
	Rev.	CATCTTGTCTGGCGAGAACT	
*ctl-2*	For.	GGACGGAAAGGCTATCTATGTG	
	Rev.	ATAGTCTGGGTCCGAAGAGG	
*ftn-1*	For.	TCATGGAATCGCCGAACAA	
	Rev.	TGCAATGTAGCGAGCAAATTC	
*smf-3*	For.	AGTTCAGGTCGAAGTTGAAGATAC	
	Rev.	CCAGGTAGGCGATTGACATTAG	
*ama-1* [107]	For.	TGGAACTCTGGAGTCACACC	
	Rev.	CATCCTCCTTCATTGAACGG	
*mito-1* [107]	For.	GTTTATGCTGCTGTAGCGTG	
	Rev.	CTGTTAAAGCAAGTGGACGAG	

#### Mitochondrial DNA quantification

Relative mtDNA content was quantified via quantitative real time PCR as previously described [107]. In brief, 25 L4 animals were collected and lysed in 10μL lysis buffer. Quantitative PCR was performed as described for qRT-PCR. mtDNA was measured with *mito-1* primers and normalized to nuclear DNA measured with primers for *ama-1* (Table 6). The ratio of mtDNA to nuclear DNA was used to generate the relative mtDNA content of all samples.

#### 2,2’-Bipyridyl treatment

L1 animals were plated on MYOB plates with OP50 bacteria. After 24 hours, animals were transferred to plates containing Vehicle (EtOH; 01.% v:v) or 100μM 2–2’-BP (in EtOH; 0.1% v:v; Millipore Sigma #D216305). Following 16 hours of exposure to 2–2’-BP or Veh., samples were processed, RNA was extracted and cDNA was synthesized as described above.

#### Ferric ammonium citrate (FAC) treatment

L1 animals were plated on MYOB plates containing Vehicle (H_2_O; 1%v:v) or 5mg/mL FAC (in H_2_O; 1% v:v) and collected for RNA extraction 48 hours after plating. Plates were seeded with OP50 bacteria. Samples were processed, RNA was extracted and cDNA was synthesized as described above.

### Statistical analysis

All data represented as mean ± standard deviation unless otherwise stated. Statistical analyses were performed with Graphpad Prism 9 software. Specific statistical tests varied depending on analysis and are indicated in figure legends.

## Supporting information

S1 Fig Analysis of *nfu-1* gene expression in *nfu-1* mutants. Gene expression analysis of *nfu-1* in *nfu-1* mutants (n = 4–6). No statistically significant changes.
(TIF)

S2 Fig Altered development and fecundity of *nfu-1* knockdown or mutants. (A-C) Developmental stage of animals exposed to EV or *nfu-1* RNAi at 24 (A), 48 (B), or 72 (C) hours after removing the P_0_ animal (n = 25–29). Young adult stage determined as post-L4 but without embryos. (D) Viability of animals exposed to EV or *nfu-1* RNAi (n = 25–29). (E) Progeny laid per day for WT and *nfu-1* mutants (n = 11–15). Only those mutants that laid embryos included. Each point represents an individual animal. (F) Representative TUNEL labeling of L4 germlines for double strand DNA breaks. Blue: DAPI; Green: TUNEL. Scale bar: 100 pixels. (G) Tissue-specific *nfu-1* knockdown effect on sterility (n = 10–11). NL2098: germline-only knockdown; VP303: intestine-only knockdown; AMJ345: germline- and intestine-only knockdown; NR350: muscle-only knockdown. A-C; G: **:p≤0.01; ***:p≤0.001; ****:p≤0.0001 by parallel two-tailed Student’s T-test with Holm-Sidak correction for multiple comparisons. D: ****: p≤0.0001 by Two-way ANOVA with Sidak correction for multiple comparisons. EV: empty vector.
(TIF)

S3 Fig Details of mitochondrial physiology assessments. (A) OCR traces for each *nfu-1* mutant (blue traces) against WT animals (red trace) (n = 11–14). The WT trace is the same in all graphs. Addition of drugs is denoted in the top left panel. (B) Relative change in OCR from basal following addition of DCC. No statistically significant differences (n = 11–14). (C) Relative mtDNA content. mtDNA content was normalized to nuclear DNA content and values are presented as that ratio relative to WT (n = 5). (D) Average pixel intensity of the COX-4::GFP signal in hypodermis of the *nfu-1* mutants (n = 11–32). No statistically significant differences. (E) Enlarged images from Fig 3D for detail of TMRE labeling. (C) ****:p≤0.0001 between WT *nfu-1* mutants via ANOVA with Dunnet correction for multiple comparisons. DCC: N,n’-dicyclohexylcarbodiimide (ATP synthase inhibitor); FCCP: Carbonyl cyanide-*4*-(trifluoromethoxy)phenylhydrazone (proton ionophore); mtDNA: mitochondrial DNA; NaN_3_: sodium azide (Complex IV inhibitor).
(TIF)

S4 Fig Direct evidence of stress regulator activation with *nfu-1* RNAi. (A) Representative images of the *hsp-6p::GFP* transgene to show activation of the UPR^mt^ upon RNAi knockdown of EV, *spg-7* (positive control), or *nfu-1*. Images captured at 10X. Scale bar: 100μm. Insets show DIC image of field of view. (B) Representative images of the *skn-1b/c::GFP* transgene to show presence or absence of nuclear accumulation in intestinal cells upon RNAi knockdown of EV, *gsk-3* (positive control), or *nfu-1*. Images captured at 60X. Arrowheads indicating GFP+ nuclei. Autofluorescent gut granules are also visible. Scale bar: 20μm. Insets show DIC image of field of view. (C) Representative images of the *daf-16p*::*daf-16::GFP* transgene to show presence or absence of nuclear accumulation in intestinal cells upon RNAi knockdown of EV, *akt-1* (positive control), or *nfu-1*. Images captured at 10X. White arrowheads indicating strongly GFP+ nuclei; yellow arrowheads indicating moderately/weakly GFP+ nuclei. Boxes indicate region expanded below *akt-1* or *nfu-1* images. Scale bar: 100μm. Insets show DIC image of field of view. EV: empty vector; UPR^mt^: mitochondrial unfolded protein response.
(TIF)

S5 Fig Effects of supplementary iron or ferroptosis inhibition. (A) Gene expression of *sod* genes following Vehicle (H_2_O) or FAC treatment (n = 4). (B) Gene expression of *ctl* genes following FAC treatment (n = 4). (C) Progeny laid per day for WT and *nfu-1* mutants with Vehicle (DMSO) or fer-1 treatment (n = 9–10). Only those mutants that laid embryos included. Each point represents an individual animal. A-B: **:p≤0.01 by parallel two-tailed Student’s T-test with Holm-Sidak correction for multiple comparisons. FAC: ferric ammonium citrate.
(TIF)

S6 Fig Embryo laying pattern with NAC treatment. (A) Progeny laid per day for WT and *nfu-1* mutants with Veh. (H_2_O) or NAC treatment (n = 9–10). Only those mutants that laid embryos included. Each point represents an individual animal. Veh.: Vehicle; NAC: N-acetyl-L-cysteine.
(TIF)

S1 Table Consolidated table of *nfu-1* mutant phenotypes. Phenotypes are arranged by broad category and order of presentation in the manuscript. Changes relative to WT are indicated by minus symbol (-) or plus symbol (+) to indicate decreases and increases, respectively. Relative magnitude of changes are indicated by increasing numbers of symbols. Only statistically significant changes are indicated. NA: not assessed; NC: no change from WT.
(XLSX)
