# Supplementary figures and images for: Allele-specific mitochondrial stress induced by Multiple Mitochondrial Dysfunctions Syndrome 1 pathogenic mutations modeled in Caenorhabditis elegans
Source: PLoS Genet. 2021 Aug 27;17(8):e1009771. doi: 10.1371/journal.pgen.1009771 (PMC8428684; doi:10.1371/journal.pgen.1009771)

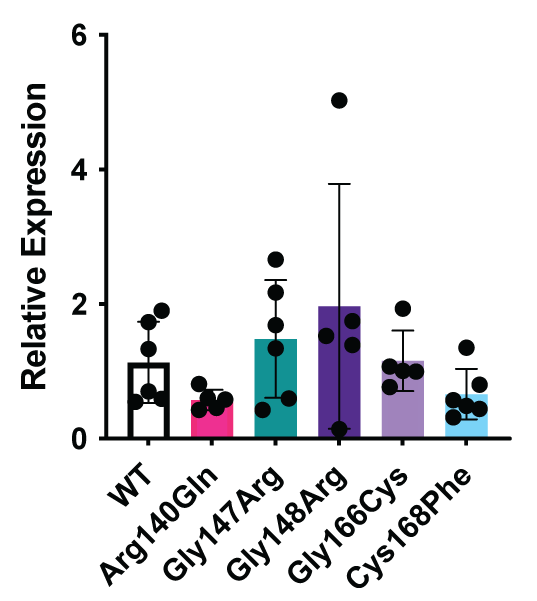

Supplement: S1 Fig — Gene expression analysis of nfu-1 in nfu-1 mutants (n = 4–6). No statistically significant changes. (TIF) [file pgen.1009771.s001.tif]

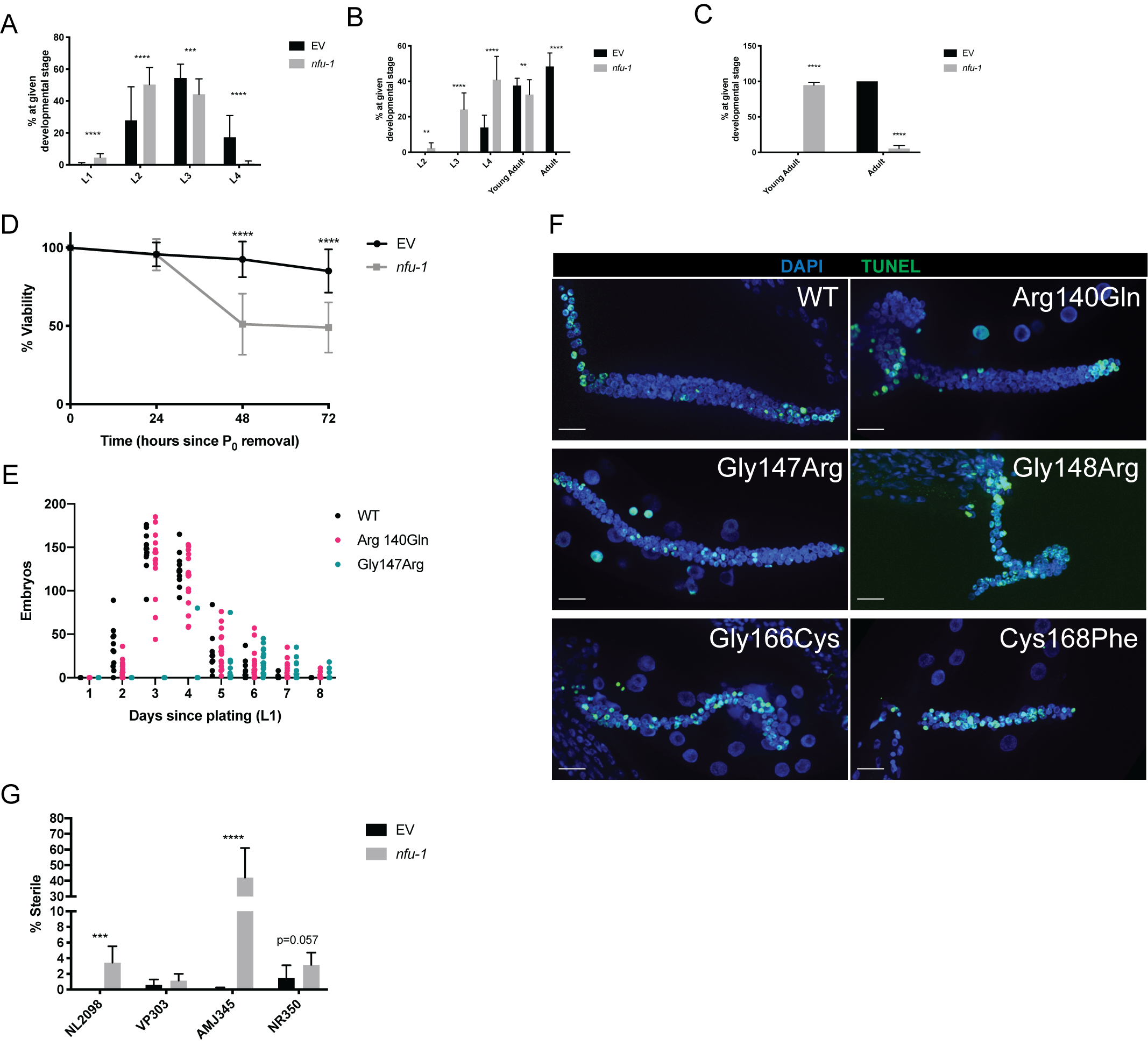

Supplement: S2 Fig — (A-C) Developmental stage of animals exposed to EV or nfu-1 RNAi at 24 (A), 48 (B), or 72 (C) hours after removing the P0 animal (n = 25–29). Young adult stage determined as post-L4 but without embryos. (D) Viability of animals exposed to EV or nfu-1 RNAi (n = 25–29). (E) Progeny laid per day for WT and nfu-1 mutants (n = 11–15). Only those mutants that laid embryos included. Each point represents an individual animal. (F) Representative TUNEL labeling of L4 germlines for double strand DNA breaks. Blue: DAPI; Green: TUNEL. Scale bar: 100 pixels. (G) Tissue-specific nfu-1 knockdown effect on sterility (n = 10–11). NL2098: germline-only knockdown; VP303: intestine-only knockdown; AMJ345: germline- and intestine-only knockdown; NR350: muscle-only knockdown. A-C; G: **:p≤0.01; ***:p≤0.001; ****:p≤0.0001 by parallel two-tailed Student’s T-test with Holm-Sidak correction for multiple comparisons. D: ****: p≤0.0001 by Two-way ANOVA with Sidak correction for multiple comparisons. EV: empty vector. (TIF) [file pgen.1009771.s002.tif]

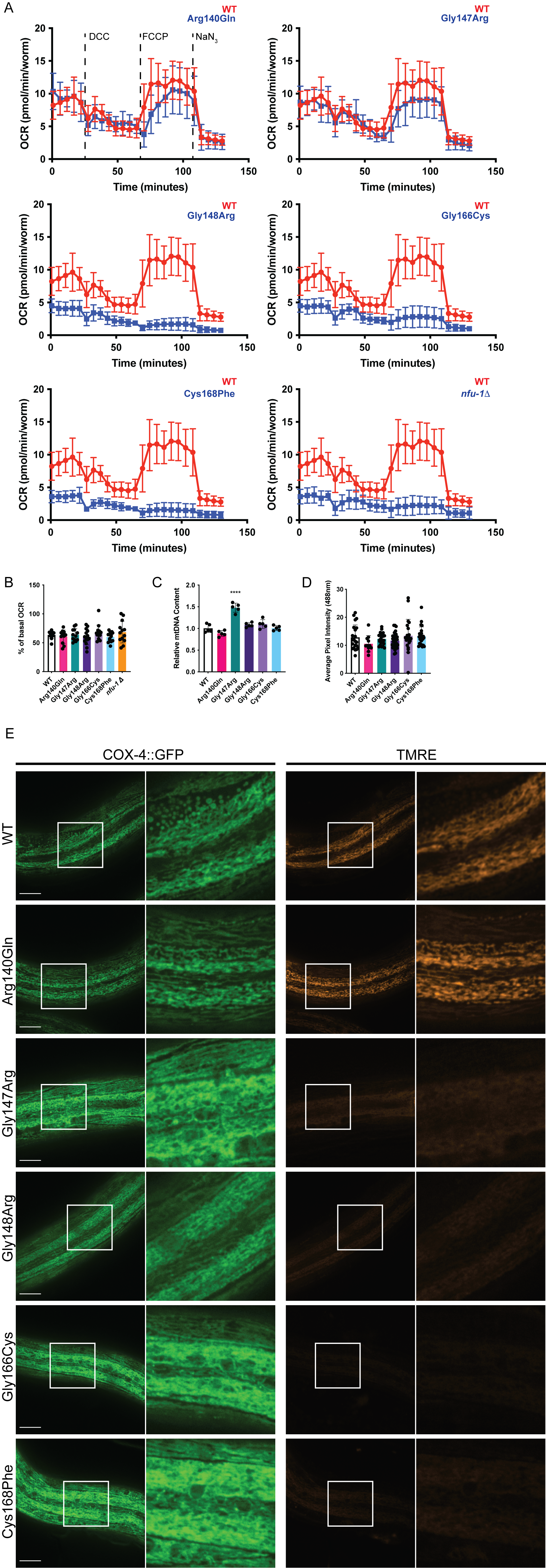

Supplement: S3 Fig — (A) OCR traces for each nfu-1 mutant (blue traces) against WT animals (red trace) (n = 11–14). The WT trace is the same in all graphs. Addition of drugs is denoted in the top left panel. (B) Relative change in OCR from basal following addition of DCC. No statistically significant differences (n = 11–14). (C) Relative mtDNA content. mtDNA content was normalized to nuclear DNA content and values are presented as that ratio relative to WT (n = 5). (D) Average pixel intensity of the COX-4::GFP signal in hypodermis of the nfu-1 mutants (n = 11–32). No statistically significant differences. (E) Enlarged images from Fig 3D for detail of TMRE labeling. (C) ****:p≤0.0001 between WT nfu-1 mutants via ANOVA with Dunnet correction for multiple comparisons. DCC: N,n’-dicyclohexylcarbodiimide (ATP synthase inhibitor); FCCP: Carbonyl cyanide-4-(trifluoromethoxy)phenylhydrazone (proton ionophore); mtDNA: mitochondrial DNA; NaN3: sodium azide (Complex IV inhibitor). (TIF) [file pgen.1009771.s003.tif]

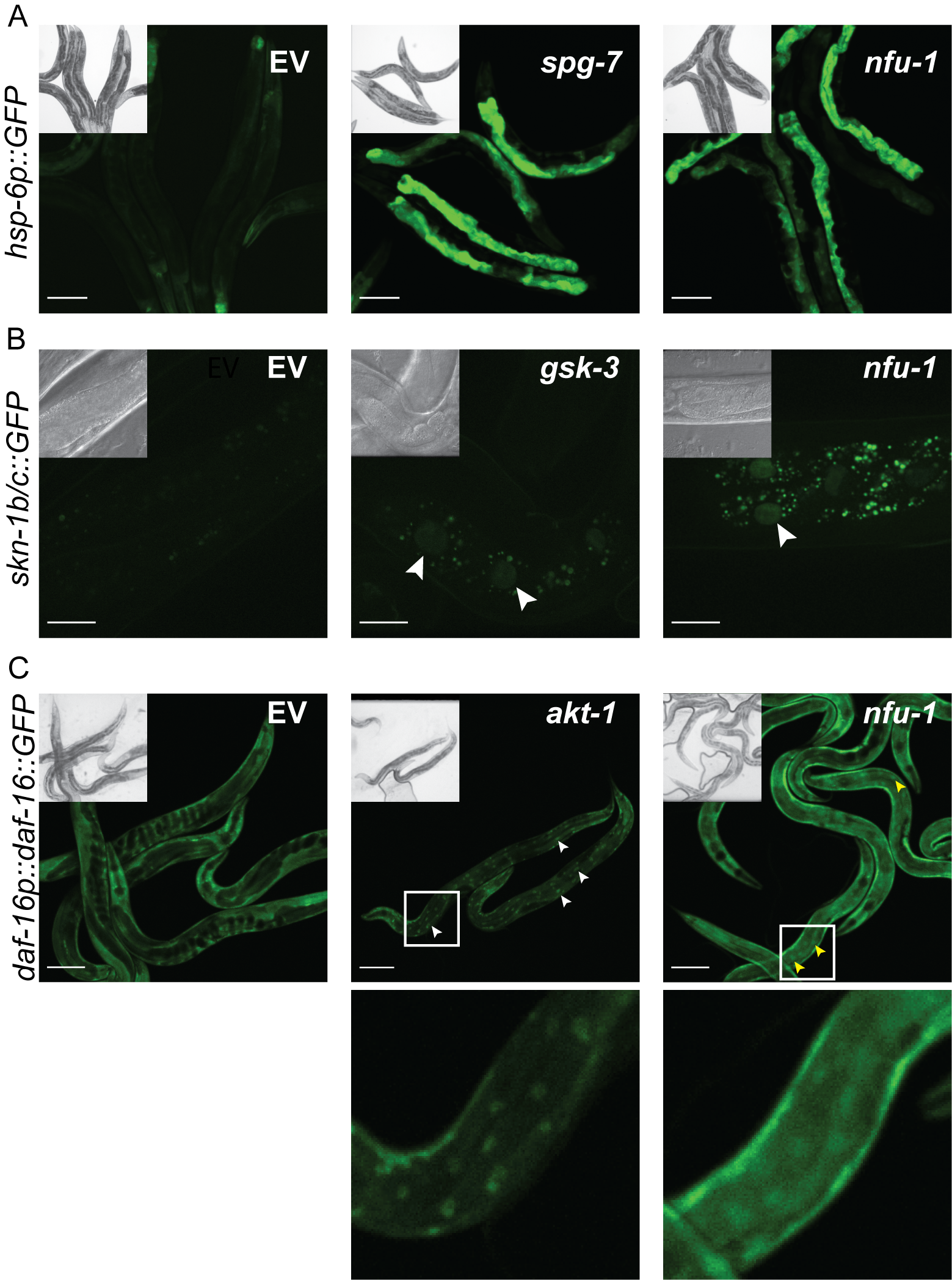

Supplement: S4 Fig — (A) Representative images of the hsp-6p::GFP transgene to show activation of the UPRmt upon RNAi knockdown of EV, spg-7 (positive control), or nfu-1. Images captured at 10X. Scale bar: 100μm. Insets show DIC image of field of view. (B) Representative images of the skn-1b/c::GFP transgene to show presence or absence of nuclear accumulation in intestinal cells upon RNAi knockdown of EV, gsk-3 (positive control), or nfu-1. Images captured at 60X. Arrowheads indicating GFP+ nuclei. Autofluorescent gut granules are also visible. Scale bar: 20μm. Insets show DIC image of field of view. (C) Representative images of the daf-16p::daf-16::GFP transgene to show presence or absence of nuclear accumulation in intestinal cells upon RNAi knockdown of EV, akt-1 (positive control), or nfu-1. Images captured at 10X. White arrowheads indicating strongly GFP+ nuclei; yellow arrowheads indicating moderately/weakly GFP+ nuclei. Boxes indicate region expanded below akt-1 or nfu-1 images. Scale bar: 100μm. Insets show DIC image of field of view. EV: empty vector; UPRmt: mitochondrial unfolded protein response. (TIF) [file pgen.1009771.s004.tif]

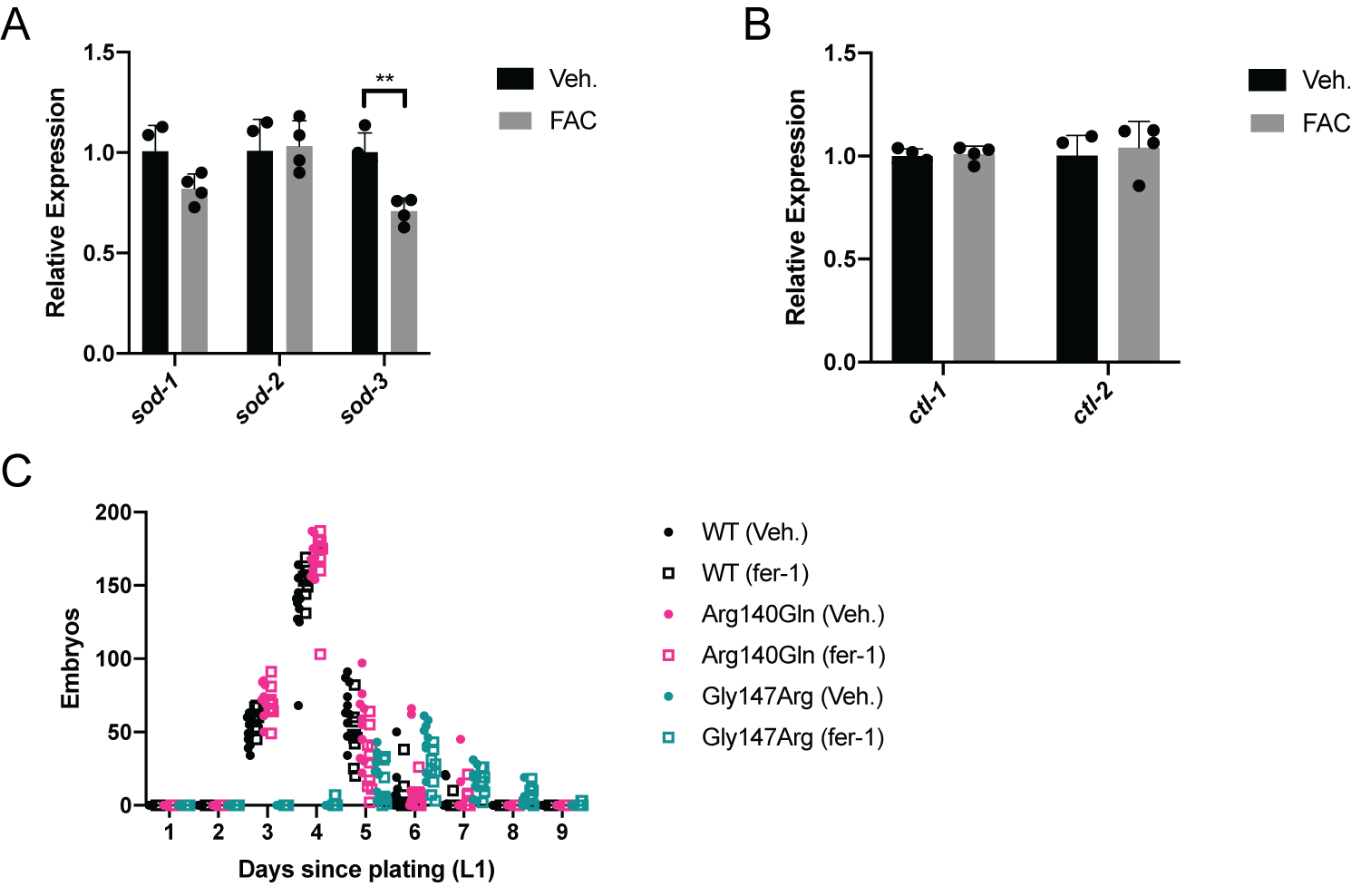

Supplement: S5 Fig — (A) Gene expression of sod genes following Vehicle (H2O) or FAC treatment (n = 4). (B) Gene expression of ctl genes following FAC treatment (n = 4). (C) Progeny laid per day for WT and nfu-1 mutants with Vehicle (DMSO) or fer-1 treatment (n = 9–10). Only those mutants that laid embryos included. Each point represents an individual animal. A-B: **:p≤0.01 by parallel two-tailed Student’s T-test with Holm-Sidak correction for multiple comparisons. FAC: ferric ammonium citrate. (TIF) [file pgen.1009771.s005.tif]

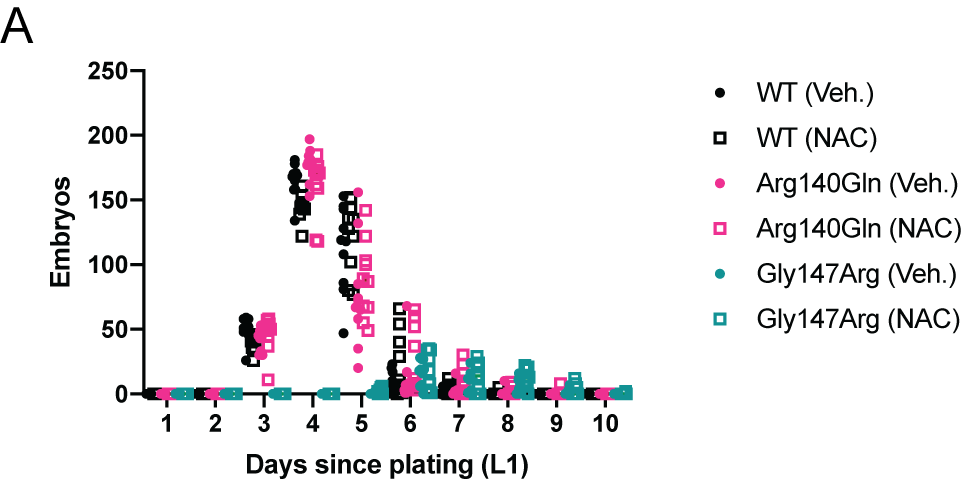

Supplement: S6 Fig — (A) Progeny laid per day for WT and nfu-1 mutants with Veh. (H2O) or NAC treatment (n = 9–10). Only those mutants that laid embryos included. Each point represents an individual animal. Veh.: Vehicle; NAC: N-acetyl-L-cysteine. (TIF) [file pgen.1009771.s006.tif]
